# Comparative Analysis of Complete Chloroplast Genomes of 13 Species in *Epilobium*, *Circaea*, and *Chamaenerion* and Insights Into Phylogenetic Relationships of Onagraceae

**DOI:** 10.3389/fgene.2021.730495

**Published:** 2021-11-04

**Authors:** Yike Luo, Jian He, Rudan Lyu, Jiamin Xiao, Wenhe Li, Min Yao, Linying Pei, Jin Cheng, Jinyu Li, Lei Xie

**Affiliations:** ^1^ School of Ecology and Nature Conservation, Beijing Forestry University, Beijing, China; ^2^ Beijing Engineering Research Center for Landscape Plant, Beijing Forestry University Forest Science Co. Ltd., Beijing, China; ^3^ College of Biological Sciences and Technology, Beijing Forestry University, Beijing, China; ^4^ Beijing Institute of Landscape Architecture, Beijing, China

**Keywords:** chloroplast genome, inversion, Onagraceae, phylogeny, RNA editing, biparental inheritance, IR expansion

## Abstract

The evening primrose family, Onagraceae, is a well defined family of the order Myrtales, comprising 22 genera widely distributed from boreal to tropical areas. In this study, we report and characterize the complete chloroplast genome sequences of 13 species in *Circaea*, *Chamaenerion*, and *Epilobium* using a next-generation sequencing method. We also retrieved chloroplast sequences from two other Onagraceae genera to characterize the chloroplast genome of the family. The complete chloroplast genomes of Onagraceae encoded an identical set of 112 genes (with exclusion of duplication), including 78 protein-coding genes, 30 transfer RNAs, and four ribosomal RNAs. The chloroplast genomes are basically conserved in gene arrangement across the family. However, a large segment of inversion was detected in the large single copy region of all the samples of *Oenothera* subsect. *Oenothera*. Two kinds of inverted repeat (IR) region expansion were found in *Oenothera*, *Chamaenerion*, and *Epilobium* samples. We also compared chloroplast genomes across the Onagraceae samples in some features, including nucleotide content, codon usage, RNA editing sites, and simple sequence repeats (SSRs). Phylogeny was inferred by the chloroplast genome data using maximum-likelihood (ML) and Bayesian inference methods. The generic relationship of Onagraceae was well resolved by the complete chloroplast genome sequences, showing potential value in inferring phylogeny within the family. Phylogenetic relationship in *Oenothera* was better resolved than other densely sampled genera, such as *Circaea* and *Epilobium*. Chloroplast genomes of *Oenothera* subsect. *Oenothera*, which are biparental inheritated, share a syndrome of characteristics that deviate from primitive pattern of the family, including slightly expanded inverted repeat region, intron loss in *clp*P, and presence of the inversion.

## Introduction

Chloroplast is one of the most important organelles in plant cells and play vital metabolic roles in photosynthesis as well as amino acid and lipid synthesis (Daniell et al., 2016). It has its own genetic material that does not obey the Mendelian laws of heredity. The chloroplast genome of angiosperms often shows a stable quadripartite ring structure containing one large single copy (LSC) region and one small single copy (SSC) region separated by two copies of an inverted repeat (IR) region. It usually shows uniparental inheritance (Ravi et al., 2008), and its sequence, gene number, and gene order have been considered to be very conserved (Wolfe et al., 1987).

However, many types of mutation occur in the chloroplast genome, including single nucleotide polymorphisms (SNPs), indels, IR contraction and expansion, inversion, and translocation (Ahmed et al., 2012; Daniell et al., 2016; Liu et al., 2018; He et al., 2019; Mehmood et al., 2020), which provide potential molecular markers for phylogenetic inference, DNA barcoding, and population genetics. Studies have shown that environmental factors, such as hot, desiccation, and metal ion stress, may have an important influence on molecular evolution (such as change GC content, promote nucleotide substitution, and decrease the abundance of small RNAs) and diversification of the plant chloroplast genomes (Fitzgerald et al., 2011; Wang et al., 2011; Ivanova et al., 2017; Gao et al., 2018; Li et al., 2020). In recent years, the use of complete chloroplast genome data for phylogenetic inference has greatly deepened our insight into the evolution of plants at a wide range of taxonomic levels (Park et al., 2017; Wen et al., 2018; Li et al., 2019; Valcárcel and Wen, 2019; Wang L. et al., 2020; Brandrud et al., 2020).

The inheritance of chloroplast genomes is predominantly maternal in angiosperms. However, biparental transmission of chloroplast genome has arisen in multiple lineages of angiosperms (Hu et al., 2008). It has been estimated that approximately 20% of angiosperm species potentially have biparentally inherited chloroplast genomes (Corriveau and Coleman, 1988; Zhang et al., 2003; Zhang and Sodmergen, 2010). Biparental inheritance of chloroplast may have important impact on evolution, such as producing genetic incompatibility to arise in speciation (Greiner et al., 2011). It has also been hypothesized that the nature of chloroplast inheritance may affect its genome stability (Wicke et al., 2011). Although the underlying mechanisms are unknown, structural rearrangements in chloroplast genome in correlation with biparental inheritance had been recognized in various kinds of plant taxa (Jansen and Ruhlman, 2012; Choi et al., 2020).

The evening primrose family, Onagraceae, is composed of about 650 species of herbs, shrubs, and rarely trees distributed worldwide and species-rich in the New World (Raven, 1988). Onagraceae is characterized by flowers with four (or rarely two or five) petals, an inferior ovary, an often dehiscent capsule, and pollen grains held together by viscin threads. The family was sharply defined (Raven, 1964), but with disputed interpretation of subfamily, tribal, and some generic delimitation in its long taxonomic history (Kurabayashi et al., 1962; Raven, 1964; Munz, 1965; Wagner et al., 2007). Several molecular phylogenetic analyses using Sanger’s sequencing method have been conducted to resolve the phylogenetic relationships within Onagraceae (Martin and Dowd, 1986; Crisci et al., 1990; Bult and Zimmer, 1993; Conti et al., 1993; Levin et al., 2003, 2004; Berry et al., 2004; Hoggard et al., 2004; Evans et al., 2005; Ford and Gottlieb, 2007; Xie et al., 2009; Liu et al., 2017). Based on molecular and morphological data, a recent taxonomic monograph by Wagner et al. (2007) included 22 genera in Onagraceae. These genera were further grouped into two subfamilies: subfam. Ludwigioideae W. L. Wagner and Hoch (with only one genus, *Ludwigia* L.) and subfam. Onagroideae W. L. Wagner and Hoch (with six tribes and 21 genera). Onagraceae contains many popular garden plants including evening primrose (*Oenothera* L.) and fuchsia (*Fuchsia* L.). Some species of the family also have medicinal value and are widely used to make oil, spices, and nectar (Chen et al., 2007).

Inheritance of the chloroplast genome in Onagraceae has attracted great attention of botanists (Cleland, 1972; Chiu et al., 1988; Chiu and Sears, 1992; Chiu and Sears, 1993; Sears et al., 1996; Massouh et al., 2016; Sobanski et al., 2019). Both maternal and biparental inheritance of chloroplast genomes has been reported in the family (Wagner et al., 2007). *Oenothera* subsect. *Oenothera* are known to have biparentally transmitted chloroplast (Corriveau and Coleman, 1988; Wagner et al., 2007). Whereas, chloroplast genomes from *Circaea* L. and *Fuchsia* have been shown to be maternally transmitted (Corriveau and Coleman, 1988; Zhang et al., 2003). Chloroplasts of *Epilobium* L. were also reported to be mainly maternally transmitted, but very low proportions of paternally transmitted chloroplast were also found (Schmitz and Kowallik, 1986). As mentioned above, biparentally inherited chloroplast genomes of many plant taxa have shown extensive rearrangement of genome structure. Thus, Onagraceae provides an opportunity to better understand differences in the chloroplast genome structure and sequence diversification between the two inheritance types. However, there are still no comparative studies concerning this issue and only a limited number of complete chloroplast genomes have been published to date.

In the present study, we report the complete chloroplast genomes from three genera (*Circaea*, *Chamaenerion* Ség.^1^, and *Epilobium*) of Onagraceae, among which those of the *Circaea* are reported for the first time. We hypothesized that the structure and sequence variation of chloroplast genomes in Onagraceae show different structures between biparentally and maternally inherited chloroplast genomes. Thus, we compared the synteny and chloroplast genome structure across the family and investigated their chloroplast genome structure and sequence variation. We also conducted a phylogenetic study to explore the evolutionary trends of chloroplast genome variation and the potential application value of the chloroplast markers across Onagraceae.

## Materials and Methods

### Taxon Sampling and Next-Generation Sequencing

We sampled 16 accessions representing three genera (*Circaea*, *Chamaenerion*, and *Epilobium*) and 13 species of Onagraceae (Supplementary Table S1). We also retrieved all the (15 samples representing 14 species) published complete chloroplast genome sequences of Onagraceae to date, as well as two samples from Lythraceae (sister family of Onagraceae) from GenBank for phylogenetic analysis. In total, five genera (*Circaea*, *Chamaenerion*, *Epilobium*, *Ludwigia*, and *Oenothera*) and 27 species (31 samples) of Onagraceae were included in this study. The taxonomy of Onagraceae at generic and infrageneric level followed Wagner et al. (2007). Our sampling covered both subfamilies (subfam. Ludwigioideae and subfam. Onagroideae) and three of the total six tribes in subfam. Onagroideae. Biparentally inherited chloroplast genomes were known to have occurred in species of *Oenothera* subsect. *Oenothera* (Wagner et al., 2007). So, we used chloroplast genome of *O. biennis* L. as a representative of biparentally inherited chloroplast genome (also reported by Corriveau and Coleman, 1988) to compare with the maternally inherited one from *Circaea* and *Epilobium*.

Approximately 50 mg dried leaf tissue was ground for each sample. Total genomic DNA was extracted using the cetyl-trimethylammonium bromide (CTAB; Doyle and Doyle, 1987) method. The quality of DNA was assessed by 0.8% agarose gel electrophoresis, and extracted DNA was sent to Novogene (http://www.novogene.com, China) for short-insert (350 bp) library construction and next-generation sequencing. Paired-end reads of 2 × 150 bp were generated on the Illumina Hiseq 4,000 Genome Analyzer platform. We used the FASTX Toolkit (http://hannonlab.cshl.edu/fastx_toolkit) to filter the raw reads and remove the adaptors and low-quality reads to obtain high-quality data. The BLAT analysis, as implemented in a Python script (Weitemier et al., 2014), was applied to exclude nuclear and mitochondrial reads using a published complete chloroplast genome sequence of *Epilobium ulleungensis* as the reference (GenBank accession no. MH198310). Subsequently, the putative chloroplast reads were *de novo* assembled using Geneious v. Prime (Kearse et al., 2012) with a low sensitivity setting. Gaps between contigs were filled by re-mapping the entire reads to both contigs using the FineTuning program in Geneious v. Prime (iterating up to 100 times), as described by He et al. (2019). Contigs were connected into larger contigs by overlapping their terminal sequences using the RepeatFinder option in Geneious v. Prime (Kearse et al., 2012). After building an approximate 130-kb contig (including a complete SSC, a complete IRa, a complete LSC, and a partial IRb region) for each sample, the boundaries of the IR region were determined using the RepeatFinder. The IR region was manually inverted and duplicated to construct the complete chloroplast genome sequence using Geneious v. Prime (Kearse et al., 2012). The correction of the gaps and junctions between IRs and LSC/SSC regions were confirmed by PCR amplifications. The complete chloroplast genome sequences were annotated using the Plastid Genome Annotator (Qu et al., 2019) and checked manually in Geneious v. Prime (Kearse et al., 2012). Illustrations of the newly sequenced chloroplast genome sequences were drawn using the Organellar Genome DRAW tool v. 1.3.1 (Lohse et al., 2013).

### Comparative Evaluation of the Chloroplast Genome

The newly sequenced chloroplast genomes were compared with those of the other published Onagraceae species. Amino acid frequency and codon usage were calculated using the Geneious v. Prime (Kearse et al., 2012) and CodonW v. 1.4 (Peden, 1999) software, and the putative RNA editing sites in protein-coding genes were determined by the predictive RNA editor for plant chloroplasts (PREP-cp) suite (Mower, 2009). For the synteny analysis of the Onagraceae chloroplast genome, mVISTA (Frazer et al., 2004) was used in LAGAN and Shuffle-LAGAN mode, with default parameters using *Epilobium sikkimense* Hausskn. as reference. The contraction and expansion of the IR boundaries between the four main parts of the genome (LSC/IRb/SSC/IRa) were visualized using IRscope (Amiryousefi et al., 2018). We also conducted a sliding window analysis to identify the nucleotide variability (Pi) of the complete chloroplast genomes of the three newly sequenced genera and *Oenothera* using DnaSP v. 5 (Librado and Rozas, 2009).

The microsatellites were determined by MIcroSAtellite (MISA) (Varshney et al., 2005), with a minimum threshold of seven nucleotides for mononucleotide repeats, four for di-, and 3 each for tri-, tetra-, penta-, and hexanucleotide repeats. The REPuter program (Kurtz et al., 2001) was used to analyze forward (F), reverse (R), complement (C), and palindromic (P) oligonucleotide repeats with a minimum repeat size of 30 bp and similarities of 90%. Furthermore, tandem repeats were evaluated by the Tandem Repeats Finder (Benson, 1999) using default parameters.

### Phylogenetic Analysis

The phylogenetic analysis was performed among 31 species of Onagraceae using two Lythraceae samples as outgroups. For phylogenetic tree reconstruction, we removed IRa from the analysis and manually reverted the inverted regions in samples of *Oenothera* subsect. *Oenothera*. We also divided the complete chloroplast genome sequences into coding regions (CDs, including protein-coding genes, tRNA genes, and rRNA genes), intergenic spacer regions (IGS), and introns. Each dataset was further divided into LSC, SSC, and IR regions. All the 13 separated and combined datasets (the complete CDs sequence, the complete IGS, the complete intron, the LSC-CDs, the LSC-IGS, the LSC intron, the SSC-CDs, the SSC-IGS, the SSC-intron, the IR-CDs, the IR-IGS, the IR-intron, and the complete chloroplast genome datasets) were then aligned using MAFFT v. 6.833 (Katoh et al., 2005) and manually adjusted by Geneious v. Prime (Kearse et al., 2012). The ambiguous alignments were removed from the datasets using a Python script (He et al., 2019).

We used both the maximum likelihood (ML) and Bayesian inference (BI) methods for phylogenetic reconstruction for each dataset. The ML tree for each dataset was generated by RAxML v.8.1.17 (Stamatakis, 2014) using the GTR + G model as suggested in the user manual. The bootstrap percentages were calculated after 500 replicates.

Bayesian inference for each dataset was conducted using MrBayes v3.2.3 (Ronquist and Huelsenbeck, 2003). Substitution models and data partitions of the complete chloroplast genome dataset for the Bayesian analysis were determined by PartitionFinder v2.1.1 (Lanfear et al., 2017). Six partitioning schemes were used for the complete chloroplast genome dataset: 1) no partitions, 2) partitioned by coding and non-coding regions (with the four rRNA genes as the third partition), 3) by LSC, SSC, and IRs, 4) coding region by genes (non-coding region as one partition), 5) coding region by genes and codon positions (non-coding region as one partition), 6) coding region by the third codon position (the first and second codon positions as on partition and the third position as the other partition, non-coding region as another one partition). The best scheme was selected according to the Bayesian information criterion (BIC). Partitioning of other datasets was on the basis of the result of the complete chloroplast genome dataset.

For the Bayesian inference, the default priors in MrBayes were applied for tree search. Two independent Markov chain Monte Carlo (MCMC) chains were created, each with three heated and one cold chain for 2,000,000 generations and sampling trees every 100 generations. The first 25% of the trees were discarded as burn-in, and the remaining trees were used to generate the consensus tree. All the alignments used in this study are available on Zenodo, with the identifier https://doi.org/10.5281/zenodo.5545914.

## Results

### Chloroplast Genome Assembly, Organization, and Nucleotide Composition Features

For each newly sampled Onagraceae species, approximately 6 Gb clean NGS data were obtained, which means that the whole genomic coverage of our NGS data ranged from ca. 6–30 × (https://cvalues.science.kew.org/). We filtered out 130,748–410,678 chloroplast reads from the samples for *de novo* assembly. The coverage of the chloroplast genome was from 79 to 271 ×. One to seven large contigs were retained. All the gaps between the *de novo* contigs were successfully bridged by re-mapping the cleaned reads to both contigs using the FineTuning program in Geneious v. Prime (Kearse et al., 2012) with 100 iterations. The correction of the gaps and junctions between IRs and LSC/SSC regions were confirmed by PCR amplifications. All the newly assembled sequences were deposited in the GenBank under accession numbers of MZ326160 and from MZ353628 to MZ353642 (Supplementary Table S1).

Chloroplast genome sequences of *Circaea* ranged from 155,817 bp (*C. alpina* subsp. *micrantha* (A. K. Skvortsov) Boufford) to 156,024 bp (*Circaea alpina* subsp. *caulescens* (Kom.) Tatew.) in size, and the overall GC content varied from 37.7 to 37.8%. For *Chamaenerion* samples, the complete chloroplast genome sequences ranged from 159,496 bp (*C. conspersum* (Hausskn.) Kitam.) to 160,416 bp (*C. angustifolium* subsp. *circumvagum* (Mosquin) Moldenke), and the overall GC content varied from 38.1 to 38.2%. For *Epilobium* chloroplast genome, the sizes ranged from 160,748 bp (*Epilobium amurense* subsp. *amurense* Hausskn.) to 161,144 bp (*E. sikkimense* Hausskn.), and the overall GC content varied from 38.1 to 38.2% (Supplementary Table S2).

**FIGURE 1:**
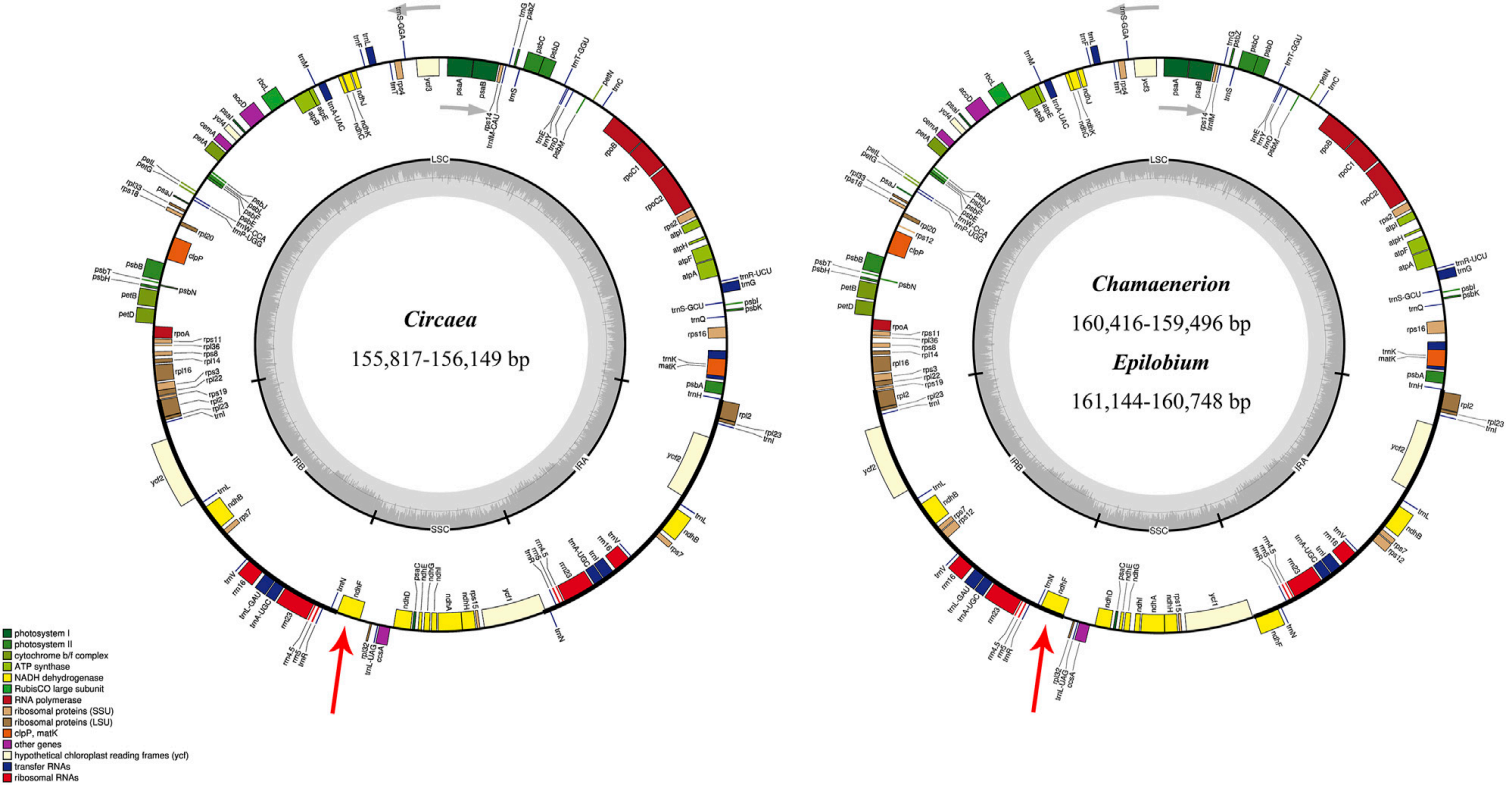
Chloroplast genome maps of *Chamaenerion*, *Circaea*, and *Epilobium* sampled in the present study. Thick lines on the outer circle identify inverted repeat regions (IRa and IRb). The innermost track indicates the G + C content. Genes on the outside of the map are transcribed in a clockwise direction, and genes on the inside of the map are transcribed in a counterclockwise direction. IR, inverted repeat; LSC, large single copy; SSC, small single copy. Red arrows showed the different IR-SC boundaries between the two chloroplast genome structures.

All the newly assembled chloroplast genome sequences contained a pair of IRs (24,996–27,519 bp) separated by a LSC region (87,569–89,163 bp) and a SSC region (17,157–18,283 bp). The complete chloroplast genomes encoded an identical set of 112 genes, including 78 protein-coding genes, 30 transfer RNAs, and four ribosomal RNAs. Among these, 17 (in *Circaea* samples) and 18 (in *Chamaenerion* and *Epilobium* samples) genes were duplicated in IR, and 18 genes had introns (Figure 1; Table 1, and Supplementary Table S2). Among the 18 intron-containing genes, 16 (10 protein-coding genes and 6 tRNA genes) had one intron and two (*ycf*3 and *clp*P) had two introns. However, the two introns in *clp*P gene are absent in *Oenothera* sect. *Oenothera* samples. The longest intron (2,487 bp) was in the *trn*K gene of *Epilobium williamsii* P. H. Raven.

**TABLE 1:** Genes present in the chloroplast genome of the 16 newly sequenced *Epilobium*, *Circaea*, and *Chamaenerion* samples.

Gene type	Gene name				
Ribosomal RNA genes	16S rRNA	23S rRNA	4.5S rRNA	5S rRNA	
Transfer RNA genes	*trn*A-UAC gene	*trn*A-UGC gene	*trn*C gene	*trn*D gene	*trn*E gene
	*trn*F gene	*trn*fM gene	*trn*G-UCC gene	*trn*G-GCC gene	*trn*H gene
	*trn*I gene	*trn*K gene	*trn*L-UAA gene	*trn*L-CAA gene	*trn*L-GAU gene
	*trn*L-UAG gene	*trn*M gene	*trn*N gene	*trn*P-UGG gene	*trn*Q gene
	*trn*R gene	*trn*R-UCU gene	*trn*S gene	*trn*S-GCU gene	*trn*S-GGA gene
	*trn*T-UGU gene	*trn*T-GGU gene	*trn*V gene	*trn*W-CCA gene	*trn*Y gene
Small subunit of the ribosome	*rps*2 gene	*rps*3 gene	*rps*4 gene	*rps7 gene*	*rps8 gene*
	*rps*11 gene	*rps*12 gene	*rps*14 gene	*rps*15 gene	*rps*16 gene
	*rps*18 gene	*rps*19 gene			
The large subunit of the ribosome	*rp*l2 gene	*rpl*14 gene	*rpl*16 gene	*rpl*20 gene	*rpl*22 gene
	*rpl*23 gene	*rpl*32 gene	*rpl*33 gene	*rpl*36 gene	
RNA polymerase subunits	*rpo*A gene	*rpo*B gene	*rpo*C1 gene	*rpo*C2 gene	
NADH dehydrogenase	*ndh*A gene	*ndh*B gene	*ndh*C gene	*ndh*D gene	*ndh*E gene
	*ndh*F gene	*ndh*G gene	*ndh*H gene	*ndh*I gene	*ndh*J gene
	*ndh*K gene				
Photosystem Ⅰ	*psa*A gene	*psa*B gene	*psa*C gene	*psa*I gene	*psa*J gene
Cytochrome b/f complex	*pet*A gene	*pet*B gene	*pet*D gene	*pet*G gene	*pet*L gene
	*pet*N gene				
ATP synthase	*atp*A gene	*atp*B gene	*atp*E gene	*atp*F gene	*atp*H gene
	*atp*I gene				
Large subunit of rubisco	*rbc*L gene				
Maturase	*mat*K gene				
Protease	*clp*P gene				
Envelope membrane protein	*cem*A gene				
Subunit of acetyl-CoA-carboxylase	*acc*D gene				
Photosystem Ⅱ	*psb*A gene	*psb*B gene	*psb*C gene	*psb*D gene	*psb*E gene
	*psb*F gene	*psb*H gene	*psb*I gene	*psb*J gene	*psb*K gene
	*psb*L gene	*psb*M gene	*psb*N gene	*psb*T gene	*psb*Z gene
Copper chaperone for superoxide dismutase	*ccs*A gene				
Conserved open reading frames	*ycf* 1,2,3,4				

### Codon Usage and Amino Acid Frequencies

Relative synonymous codon usage (RSCU) of the chloroplast genome sequences of the newly assembled samples was calculated using all protein-coding genes. Results of amino acid frequency, RSCU, and putative RNA editing sites are shown in Supplementary Figure S1 and Supplementary Tables S3, S4. There were 50 putative RNA editing sites detected in the 18 protein-coding genes of *Epilobium*, 43 sites detected in 16 protein-coding genes of *Circaea*, and 48 sites detected in 17 protein-coding genes of *Chamaenerion*. Among the three genera, the gene with the most RNA editing sites was *ndh*B (12 sites), and the second was *ndh*D (5 sites). The most common type of substitution in *Epilobium* was serine to leucine (26%), followed by proline to leucine (18%). This phenomenon also existed in the other two genera: the *Chamaenerion* chloroplast genome displayed 31.3% of editing sites substituted from serine to leucine, and 14.6% from proline to leucine; and the *Circaea* chloroplast genome showed 32.6% of editing sites substituted from serine to leucine, and 18.6% from proline to leucine. Among the 50 recognized RNA editing sites in *Epilobium*, 35 substitutions occurred at the second nucleotide position and 15 substitutions occurred at the first nucleotide position. Similar results were also detected in the other two genera.

### Chloroplast Genome Comparison

**FIGURE 2:**
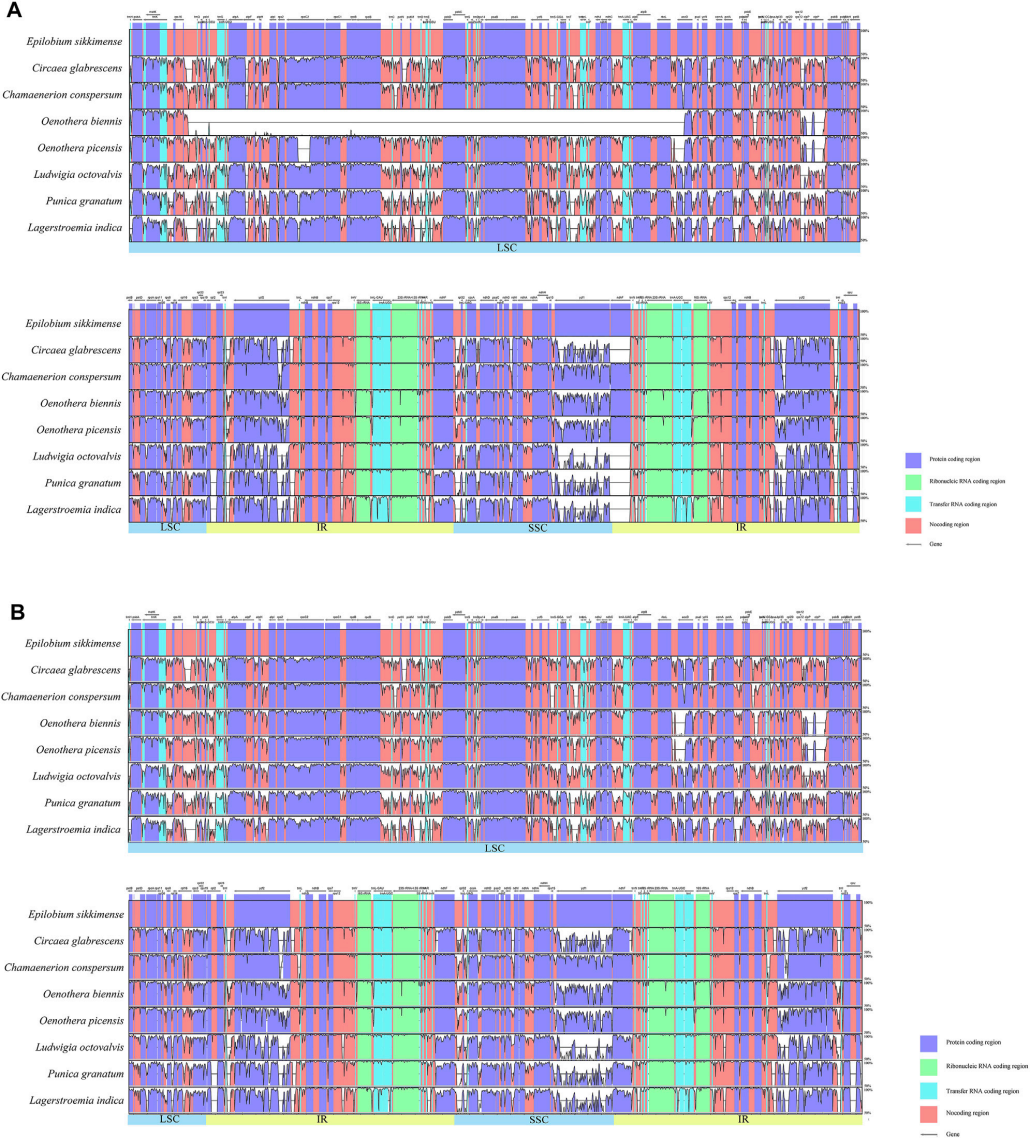
Sequence alignment of representative samples from five genera of Onagraceae and two outgroups using the mVISTA program (alignment of all 33 samples are shown in Supplementary Figure S2). A cut-off of 70% similarity was used for the plot, and the Y-scale represents the percentage similarity ranging from 50 to 100%. Blue represents coding regions, and pink represents non-coding regions. **(A)**: LAGAN method, the large empty part of the *Oenothera biennis* graph is the inverted region; **(B)**: Shuffle LAGAN method.

To investigate the synteny and structural variation of the chloroplast genomes of Onagraceae, we performed multiple alignments of all the tested samples using mVISTA (Supplementary Figure S2). LAGAN and Shuffle-LAGAN programs were applied for this analysis. Results of generic representatives are shown in Figure 2. When using the LAGAN method, *Oenothera* subsect. *Oenothera* samples showed a large area of mismatch in their LSC region due to gene inversion. This inversion occurred between *rbc*L and *trn*Q-UUG and was approximately 56 kb in length. Typically, *clp*P gene has two introns in many angiosperm species. However, these two introns are absent in *Oenothera* sect. *Oenothera* samples, but still present in *O. curtiflora* W. L. Wagner and Hoch (sect. *Gaura* (L.) W. L. Wagner and Hoch). In addition, compared with other genera, some mismatch regions were found in the IR region of *Oenothera*, *Epilobium*, and *Chamaenerion*, which was caused by expansion of their IR zones by inclusion of the *ndh*F gene, and rarely other genes (described below).

**FIGURE 3:**
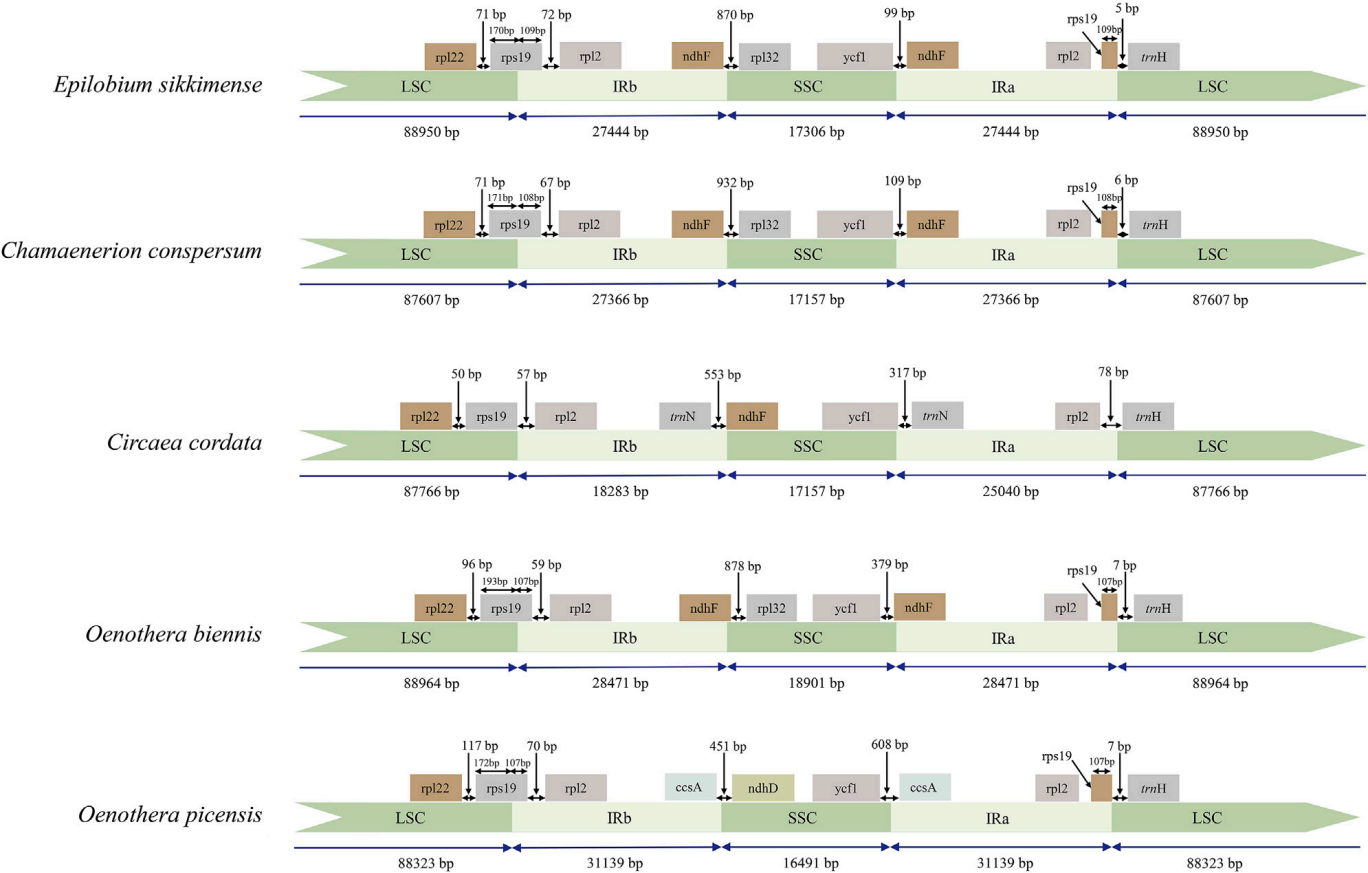
Comparison of the LSC, IR, and SSC boundary regions of representative samples the three newly sequenced genera of Onagraceae and *Oenothera* samples. IR: inverted repeats; LSC: large single copy; SSC: small single copy.

Subsequently, we compared the IR/SC boundary regions of 31 species of Onagraceae and two species of Lythraceae (Supplementary Figure S3). The early diverged genera of Onagraceae, *Ludwigia* and *Circaea*, have 17 genes in the IR region, which is the same with most other angiosperm genera such as *Amborella* Baill., *Caltha* L., and *Arabidopsis* Heynh. (Sato et al., 1999; He et al., 2019). So, the IR region of *Ludwigia* and *Circaea* can be considered as the primitive type of the family. Other tested Onagraceae genera showed more or less IR expansion. Almost all the tested samples from *Chamaenerion*, *Epilobium*, and *Oenothera* have 18-gene IR region (with inclusion of *ndh*F) (Figure 3). Two samples from *Oenothera* subsect. *Munzia* (W. Dietr.), *O. picensis* Phil. and *O. villaricae* W. Dietr., have 21-gene IR, with additional *ccs*A, *trn*L-UAG, *rpl*32 and *ndh*F genes.

**FIGURE 4:**
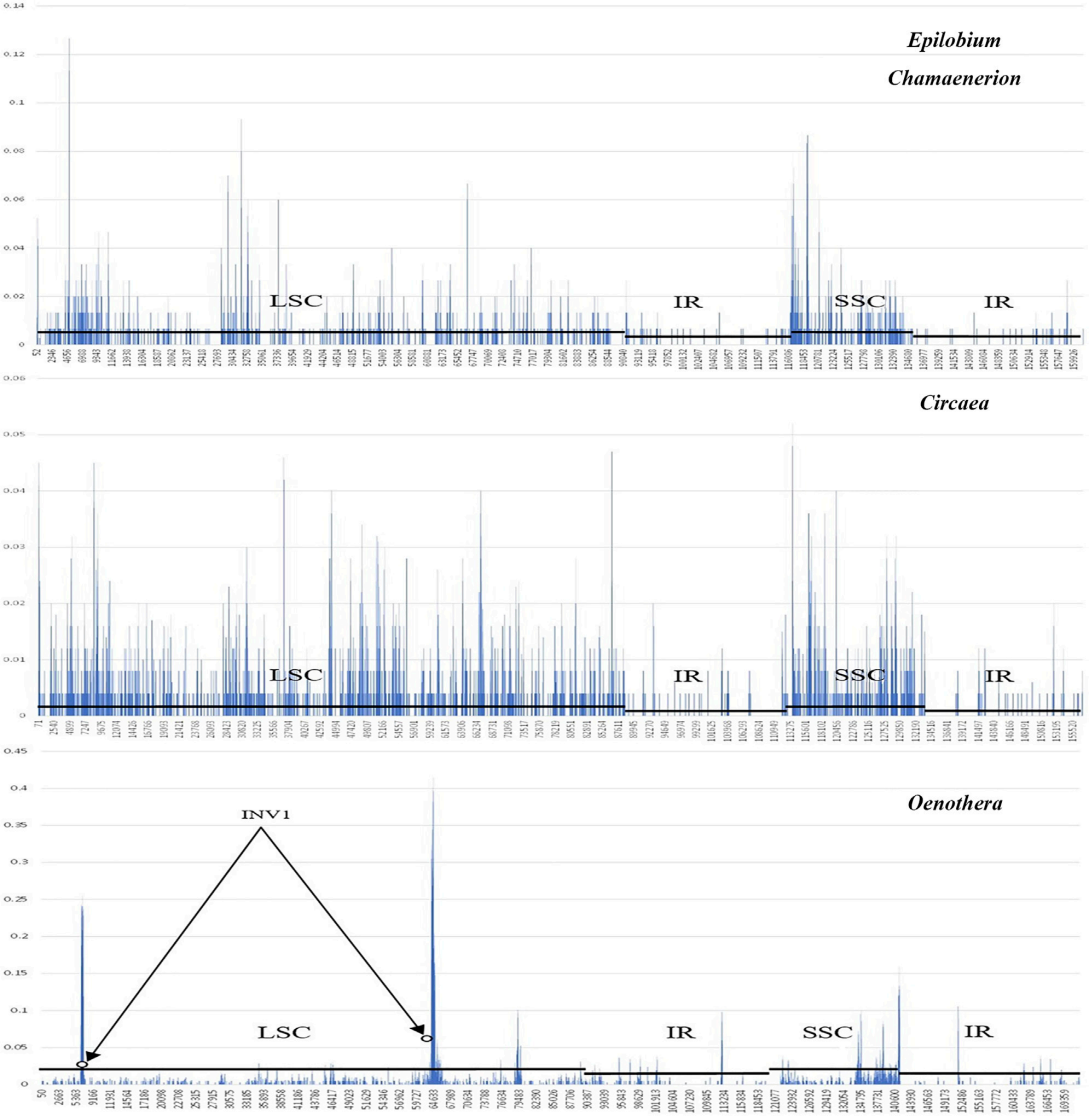
A sliding window analysis of the complete chloroplast genomes of *Epilobium*, *Chamaenerion*, *Circaea*, and *Oenothera* samples showing nulceotide variability (Pi) within each genus. Circles identify the regions bordering inversion sites of *Oenothera*, and lines parallel to the *X*-axis identify the positions of LSC, SSC, and IR regions.

Sliding window analysis (Figure 4) showed that the nuclear variability of the IR region was relatively low in the three newly sequenced Onagraceae genera as well as in published *Oenothera* samples. Among the tested genera, *Oenothera* had the highest nucleotide variation. In addition, extremely high variations were discovered at both ends of the inversion in the LSC of the *Oenothera* chloroplast genome, which may be the main cause of the structural rearrangement of chloroplast genomes in *Oenothera* subsect. *Oenothera*.

### SSR and Repeats Analyses

The chloroplast genome sequences are known to be highly conserved. However, chloroplast SSRs have been applied as important phylogenetic markers for unraveling polymorphisms across species and populations in plant molecular studies (Cato and Richardson, 1996; Xu et al., 2002; Wills et al., 2005; Bi et al., 2018). Furthermore, the primers for the chloroplast SSRs are conserved, which may facilitate primer design across species and genera. In this study, we detected 47–90 SSRs from chloroplast genome sequences of the three newly sequenced genera. Those in *Circaea* had 47–55 SSRs, which was the lowest among the three genera (Table 2). *Epilobium* and *Chamaenerion* had a higher number of SSRs, ranging from 76 to 90. The mononucleotide repeat unit (A/T) was the most common type, accounting for 85.6–97.9% of all 16 samples. The dinucleotide repeat unit (AT/TA) was the second most abundant, accounting for 1.7–9.2%. The mononucleotide repeat unit C/G existed in *Chamaenerion* and *Circaea* samples and in the chloroplast genome sequence of *Epilobium sikkimense*. *Chamaenerion* and *Epilobium* contained only two types of repeats: mononucleotide and dinucleotide. A trinucleotide repeat unit (AAT/ATT) was found in all samples of *Circaea*. Tetranucleotide repeats were found in *C. alpina* subsp. *caulescens* (AATAT/ATATA) and *C. glabrescens* (Pamp.) Hand.-Mazz. (AAAGG/AAGGA). Only one hexanucleotide repeat (AAATAT/ATAAAT) was present in *C. alpina* subsp. *caulescens*. These SSRs were mainly located in the IGS region and sometimes also occurred in introns and CDs. As expected, most SSRs were detected in the LSC region, followed by the SSC and IR regions.

**TABLE 2:** Simple sequence repeats (SSRs) for the 16 newly sequenced *Epilobium*, *Circaea*, and *Chamaenerion* samples.

Genomes	Repeat units	Number	Percentage (%)	Location			Region		
				Intron	IGS	CDS	LSC	SSC	IR
*Chamaenerion angustifolium* subsp. *circumvagum* (Mosquin) Moldenke	A/T	77	85.6	13	53	11	59	12	6
	C/G	6	6.7	2	4		4	2	
	AT/AT	7	7.8	2	5		7		
*C. angustifolium* subsp. *angustifolium* (L.) Scop.	A/T	80	90.9	14	57	9	60	14	6
	C/G	3	3.4	1	2		1	2	
	AT/AT	5	5.7	2	3		5		
*C. conspersum* (Hausskn.) Kitam.	A/T	67	87.0	7	48	12	49	10	8
	C/G	4	5.2	1	3		2	2	
	AT/AT	6	7.8	2	4		6		
*Circaea alpina* subsp. *caulescens* (Kom.) Tatew.	A/T	49	89.1	8	35	6	42	5	2
	C/G	1	1.8		1		1	1	
	AT/TA	2	3.6		2		2		
	AAT/ATA	1	1.8		1		1		
	AATAT/ATATA	1	1.8		1		1		
	AAATAT/ATAAAT	1	1.8		1				
*C. alpina* subsp. *micrantha* (A. K. Skvortsov) Boufford	A/T	41	87.2	6	29	6	33	6	2
	C/G	2	4.3	1	2		2	1	
	AT/TA	3	6.4		2		2		
	AAT/ATA	1	2.1		1		1		
*C. cordata* Royle	A/T	48	98.0	8	34	6	41	5	2
	AAT/ATA	1	2.0		1		1		
*C. glabrescens* (Pamp.) Hand.-Mazz.	A/T	53	93.0	9	38	6	46	5	2
	C/G	1	1.8		1		1		
	AT/TA	1	1.8		1		1		
	AAT/ATA	1	1.8		1		1		
	AAAGG/AAGGA	1	1.8		1		1		
*C. repens* Wall. ex Asch. & Magnus	A/T	48	92.3	5	37	6	41	5	2
	C/G	2	3.8		2		2		
	AT/AT	1	1.9		1		1		
	AAT/ATA	1	1.9		1		1		
*Epilobium amurense* subsp. *amurense* Hausskn.	A/T	75	92.6	7	62	6	58	9	8
	AT/TA	6	7.4	2	4		5	1	
*E. amurense* subsp. *cephalostigma* (Hausskn.) C.J. Chen, Hoch & P.H. Raven	A/T	77	90.6	8	63	6	61	8	8
	AT/TA	8	9.4	2	6		6	2	
*E. cylindricum* D. Don	A/T	73	92.4	8	59	6	53	12	8
	AT/TA	6	7.6	2	4		5	1	
*E. minutiflorum* Hausskn.	A/T	80	92.0	7	67	6	65	7	8
	AT/TA	7	8.0	2	5		6	1	
*E. royleanum* Hausskn.	A/T	69	90.8	7	56	6	60	7	8
	AT/TA	7	9.2	2	5		6	1	
*E. sikkimense* Hausskn.	A/T	82	96.5	7	69	6	65	9	8
	C/G	1	1.2		1		1		
	AT/TA	2	2.4		2		2		
*E. tibetanum* Hausskn.	A/T	75	92.6	8	61	6	58	9	8
	AT/TA	6	7.4	2	4		5	1	
*E. williamsii* P. H. Raven	A/T	76	92.7	7	63	6	59	9	8
	AT/TA	6	7.3	1	5		5	1	

**FIGURE 5:**
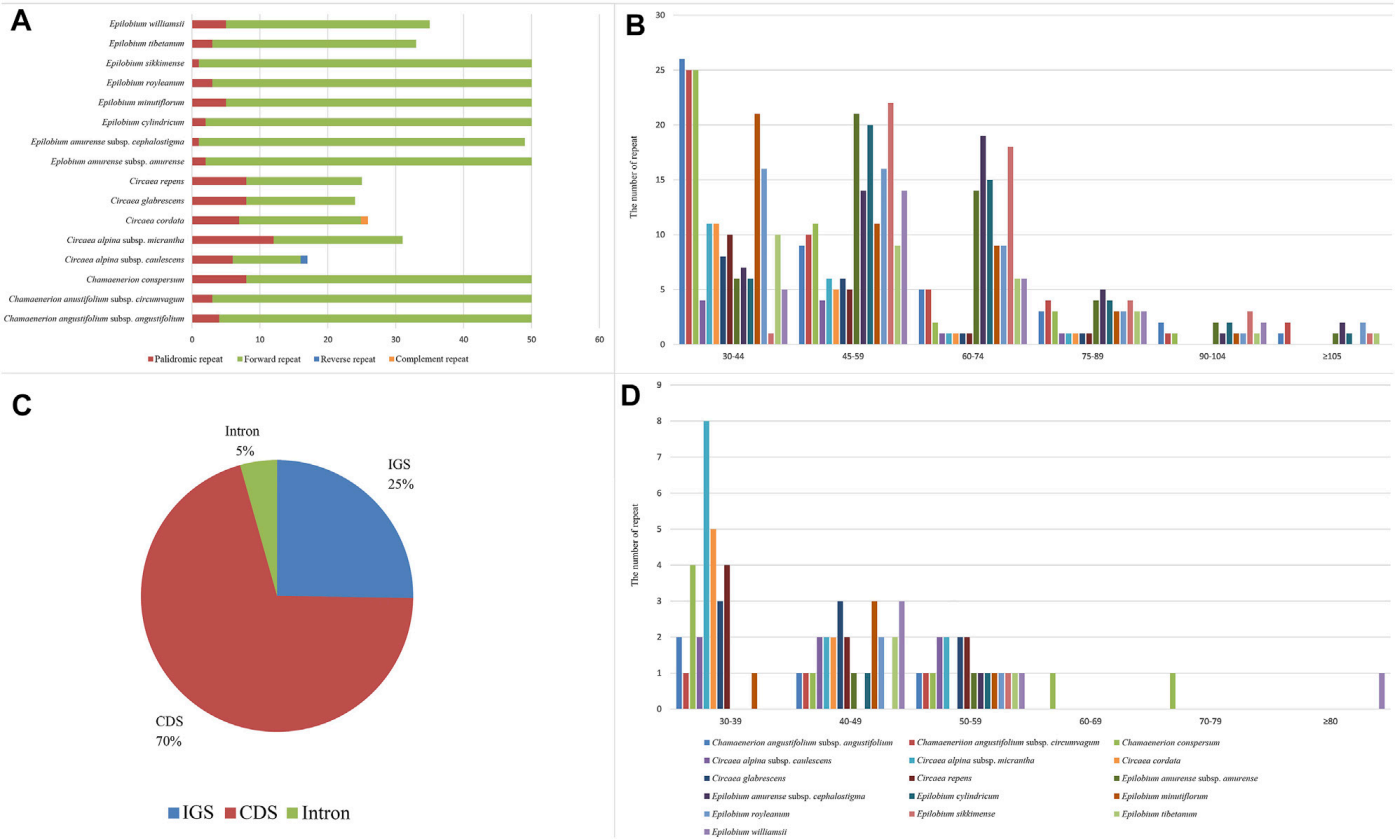
Analyses of repeated sequences in 16 newly sequenced chloroplast genomes of Onagraceae. **(A)**: number of four repeat types; **(B)**: frequency of direct repeats by length; **(C)**: location of repeats; **(D)**: frequency of palindromic repeats by length.

In addition to the SSRs, we also explored the role of repeats identified by REPuter (Kurtz et al., 2001). We found a total of 640 repeats in the 16 samples (Figure 5). Only palindromic and forward repeats were detected in *Epilobium* and *Chamaenerion*. In addition to these two types of repeat, a complement repeat was detected in *Circaea cordata* Royle, and a reverse repeat was found in *C. alpina* subsp. *caulescens*. The *Circaea* chloroplast genome sequence had 10–19 forward repeats, whereas *Chamaenerion* and *Epilobium* had 30–48 forward repeats. Among all the detected repeats, palindromic repeats accounted for 12.18% and forward repeats accounted for 87.5% of total repeats, whereas complement and reverse repeats only accounted for 0.032%. The repeat length of *Circaea* and *Chamaenerion* was shorter, and most repeats were between 30 and 44 bp. The repeat length of *Epilobium* was longer, at 30–59 bp in most samples. Much longer repeats (over 100 bp) were found in *Epilobium* and *Chamaenerion*. The longest repeat was 203 bp in length and was located in the *ycf*2 gene. The region containing the majority of the repeats was CDs (70%), followed by the IGS (25%), and the intron region (5%). A large number of repeats were found in the *ycf*2 gene (in CDs), especially in *Epilobium* and *Chamaenerion* samples. The presence of those SSRs and repeats demonstrated that the loci were potentially mutation hotspots in the chloroplast genome, and they may play an important role in developing genetic markers for future phylogenetic or population genetic studies.

### Biparentally vs. Maternally Inheritated Chloroplast Genome

**FIGURE 6:**
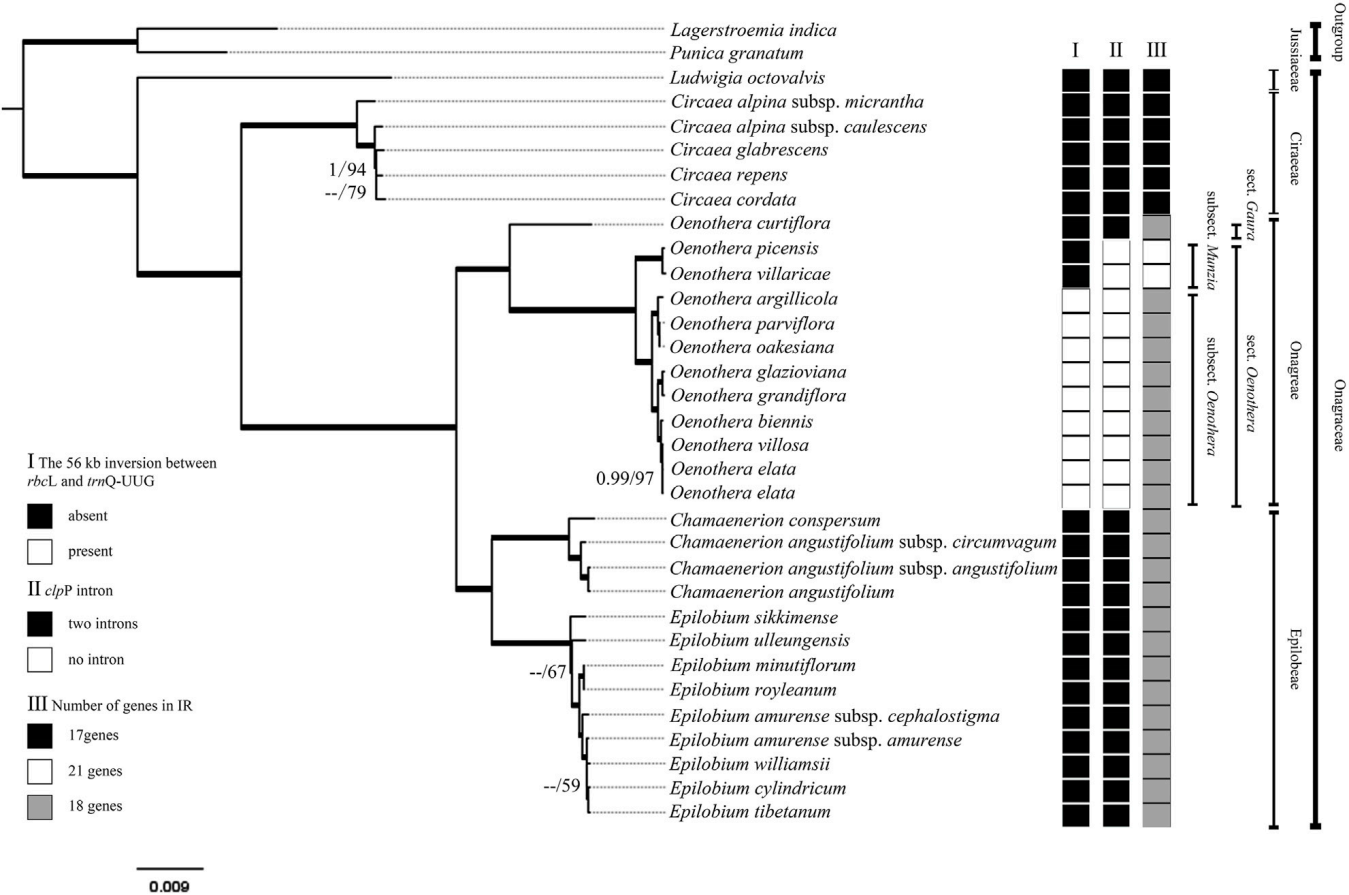
Bayesian consensus tree of Onagraceae species inferred from complete chloroplast genome sequences. Maximum likelihood (ML) bootstrap values/Posterior Probability (PP) values are shown at each node. Internal branches that are fully supported by both analyses (with 100 ML bootstrap values and 1 PP values) were thickened. ML bootstrap values < 50 and PP values < 0.95 are shown as --.

Comparing chloroplast genome sequences of *Oenothera biennis* (biparental transmitted) and *Circaea* (maternally transmitted), several structural differences are depicted. A large inversion occurs in the LSC regions of *O. biennis*, whereas chloroplast genome of *Circaea* lacks it. Two intons in *clp*P genes (present in most genera of Onagraceae) are absent in the *O. biennis*. The IR region of the *O. biennis* chloroplast genome was slightly expanded with the inclusion of *ndh*F gene (Figures 2, 3, 6). Chloroplasts of *Epilobium* were also reported to be mainly (but not entirely) maternally inherited (Schmitz and Kowallik, 1986). Chloroplast genomes of *Epilobium* samples also lack the inversion, and their *clp*P have both introns, although their IR regions were also slightly expanded*.*

Although we cannot concluded that all the members of *Oenothera* subsect. *Oenothera* have the biparentally transmitted chloroplast, the chloroplast genome structure, gene content, and the gene arrangement of all the samples from this subsection are quite stable (Figure 6). In contrast, the chloroplast genome of *Oenothera* sect. *Gaura* are the similar to those in *Chamaenerion* and *Epilobium* rather than to other *Oenothera* samples. *Oenothera* subsect. *Munzia* have *clp*P without introns, which is similar to subsect. *Oenothera*, but have much more expanded IR regions (with 21 genes) and no inversion in their chloroplast genomes.

### Phylogenetic Analysis

To better accommodate the heterogeneity of the data in the processes of Bayesian analysis, the complete chloroplast dataset was tested by six partitioning treatments. All the partitioning strategies showed similar results and no obvious improvement was observed among them. It seemed that partitioning the coding region by the third codon position obtained a little better result (Table 3). For this reason, we used this partition strategy for the datasets which have coding regions (the complete CDs sequence, the LSC-CDs, the SSC-CDs, the IR-CDs, and the complete chloroplast genome datasets), and GTR + I + G for each partition tested by PartitionFinder were applied for Bayesian analysis. We used GTR + G model tested by PartitionFinder (and no partitioning strategy) for the non-coding datasets (the complete IGS, the complete intron, the LSC-IGS, the LSC intron, the SSC-IGS, the SSC-intron, the IR-IGS, and the IR-intron) for both ML and Bayesian analyses. The complete chloroplast genome dataset (including LSC, SSC, and IR with an aligned length of 126,290 bp) generated a phylogeny (Figure 6), which is consistent with all the 12 separated phylogenies (Supplementary Figure S4).

**TABLE 3:** Comparison of partitioning strategies used for the complete chloroplast genome dataset.

Dataset	Partitioning strategy	Parameters	Subsets	ln *L*	BIC
Complete	No partition	72	1	−549020.00	1098905.52
Chloroplast	Coding and non coding	95	3	−554228.87	1109618.89
Genome	LSC, SSC, IRs	94	3	−541571.77	1084273.53
Dataset	By gene	186	12	−548690.44	1099654.32
	By gene and codon position	255	19	−545647.25	1094411.31
	By the third codon position	84	2	−528124.33	1057271.73

Phylogenies created from all the datasets using both methods were basically the same, especially for strongly supported clades. Thus in this study, our discussion was on the basis of the phylogeny inferred by the complete chloroplast genome dataset. All the Onagraceae tribes and genera were strongly supported. The genus *Ludwigia* was shown to be the first diverged genus in the Onagraceae. The phylogenetic relationship within the genus *Circaea* was not well resolved, whereas the genus *Oenothera* showed a clear phylogenetic structure. *Oenothera curtiflora* (sect. *Gaura*) was revealed to be the first diverged species in the genus. Other *Oenothera* species formed a strongly supported clade with a long branch. Within this clade, two subsections (subsect. *Munzia* and subsect. *Oenothera*) were clearly resolved with high supporting values. The genus *Epilobium* was also relatively densely sampled; however, this genus was not well resolved in the basal part of the phylogeny.

## Discussion

The present study reports the first chloroplast genomes of *Circaea*. Chloroplast genomes of some species in *Chanaenerion* and *Epilobium* were also reported for the first time. We compared the genetic diversity within each genus to obtain insight into the molecular evolution of chloroplast genomes in Onagraceae. Gene content and organization of the Onagraceae chloroplast genome were analyzed to reveal phylogenetic information pertaining to gene rearrangement. Analysis of codon usage by the chloroplast genome can aid to understand the selection pressure on genes and genome structure (Yang et al., 2014).

In the present study, the preference of codons ending with A/T in Onagraceae chloroplast genomes was confirmed (Supplementary Table S4). The same results were also observed in other angiosperm species, such as in Fabaceae, Solanaceae, Asteraceae, and many others (Nie et al., 2014; Mehmood et al., 2020; Somaratne et al., 2020). Our results also show the highest similarities of codon usage among the three newly sequenced genera (Supplementary Figure S1), indicating that these genera may have experienced similar environmental stresses in their evolutionary history. Most SSRs in the newly sequenced Onagraceae chloroplast genomes were found to be mononucleotides (A/T) (Table 2), which is similar to reports in other families of angiosperms. The genus *Circaea* contained more types of SSRs and repeats than the other two genera (Figure 5; Table 2). This SSR and repeat information may be helpful for the development of molecular markers for population genetics analysis and developing DNA barcodes.

The angiosperm chloroplast genomes are conserved in gene content and organization among different lineages (Palmer, 1985). However, structural variation and gene rearrangements in chloroplast genomes have been discovered in many angiosperm families, such as Anacardiaceae (Wang Y. B. et al., 2020), Apiaceae (Lee et al., 2019), Asteraceae (Walker et al., 2014), Campanulaceae (Haberle et al., 2008), Euporbiaceae (Tangphatsornruang et al., 2011), Geraniaceae (Weng et al., 2014), Fabaceae (Cai et al., 2008), Lentibulariaceae (Silva et al., 2019), Podostemaceae (Bedoya et al., 2019), and Ranunculaceae (Liu et al., 2018; He et al., 2019; Zhai et al., 2019). In Onagraceae, our results show the similarities in gene content and organization in chloroplast genome among almost all the sampled genera and species, with the exception of *Oenothera* subsect. *Oenothera* species that contain a large inversion (ca. 56 kb) in the LSC region (Supplementary Figure S2). This large gene inversion had been reported previously by Greiner et al. (2008) and is clearly a derived character (synapomorphy) for subsect. *Oenothera* (Figure 6). Extremely high nucleotide variations occur at both ends of this inversion, which may be the direct cause of this inversion.

The phylogenetic analysis in this study also clearly demonstrated the evolutionary trends of the other two structural variations (IR expansion and intron loss in *clp*P) of the chloroplast genome in Onagraceae. Previous studies have shown that expansion/contraction of the IR region is common in angiosperm chloroplast genomes and is the major cause of length variation in chloroplast genomes (Goulding et al., 1996; Kim and Lee, 2004). IR expansion that results in the duplication of genes has been reported in various plant taxa (Chumley et al., 2006; Lee et al., 2007; Park et al., 2017; Liu et al., 2018). Typically, there are 17 genes in the IR region in a wide range of angiosperm taxa (He et al., 2019). In Onagraceae, the early diverged genera, *Ludwigia* and *Circaea*, have 17 genes in their IR region, which may represent primitive state of this character in the family. For the other samples, two kinds of IR expansion were discovered. Chloroplast genomes of *Chamaenerion, Epilobium*, and *Oenothera* sect. *Gaura* have 18-gene IR regions, whereas, *Oenothera* subsect. *Munzia* have 21-gene IR regions. From our phylogenetic analysis, the 18-gene IR region can be seen as a derived state from the 17-gene IR regions, and the 21-gene IR region maybe further derived from the 18-gene IR region (Figure 6). The IR regions in Onagraceae seem to evolve toward gradual expansion, and no IR contraction was detected in the family by our analysis. In addition to inversion and IR expansion, another derived character, i.e., introns loss in *clp*P (Figure 6), is present in *Oenothera* sect. *Oenothera*, but not in sect. *Gaura* and other genera. In addition, the occurrence order of the three structural variations can also be inferred by our phylogenetic analysis. The IR expansion (from 17 genes to 18 genes) in *Oenothera*, *Chamaenerion*, and *Epilobium*, happened before the loss of *clp*P introns in *Oenothera* sect. *Oenothera*, and then followed by the acquisition of the large inversion in subsect. *Oenothera*.

*Oenothera* subsect. *Oenothera* seemed to be a very distinctive group carrying almost all specialized chloroplast genome variation in Onagraceae. Species of this subsection are not only known to have biparentally inherited chloroplast genomes but also known to have permanent translocation heterozygosity (PTH), a specialized system in which all seven pairs of chromosomes exchange their arms during meiosis (Cleland, 1972; Raven, 1979; Harte, 1994; Dietrich, 1997; Wagner et al., 2007). In our chloroplast genome analysis, three derived characters, presence of a large inversion, intron loss in *clp*P, and 18-gene IR, are concentrated in *Oenothera* subsect. *Oenothera*. Among them, presence of inversion is only found in this subsection. Although the presence of inversion and biparental transmission of the chloroplast genome are only possessed by *Oenothera* subsect. *Oenothera*, we still cannot tell whether biparental transmission has triggered the large inversion or vice versa, because there are many chloroplast genomes with inversions in other plant taxa (such as in Ranunculaceae, He et al., 2019) that do not have biparental plastid transmission.

The phylogenetic relationship resolved in this study is basically consistent with that reported in previous studies (Levin et al., 2003; Levin et al., 2004). However, phylogeny inferred from the complete chloroplast genome sequences was better resolved and better supported statistically than from previous studies using Sanger’s sequencing method (Bult and Zimmer, 1993; Conti et al., 1993; Levin et al., 2003; Levin et al., 2004; Sytsma et al., 2004), demonstrating that chloroplast genome sequences may be a good molecular marker for resolving phylogeny of Onagraceae at generic level. Within each genus, species phylogeny was better resolved in *Oenothera* than in *Circaea* and *Epilobium*, which is due to the higher level of variation in *Oenothera* chloroplast genome (Figure 6). This result indicates that the chloroplast genome sequences can be applied for inferring phylogenetic relationship of *Oenothera* at sectional or even species level. However, *Oenothera* has 18 sections (Wagner et al., 2007) and the chloroplast genome from only two sections have been reported. Further studies are needed to be done in the future because it is possible that the other unsampled sections might have their own distinguishing characteristics in the chloroplast genome.

## Conclusion

The complete chloroplast genome sequences of 16 samples representing 13 species in *Circaea*, *Chamaenerion*, and *Epilobium* (Onagraceae) were assembled in this study. We compared chloroplast genomes across the Onagraceae samples and obtained comprehensive molecular information including nucleotide content, codon usage, RNA editing sites, structural variation, and simple sequence repeats (SSRs) through bioinformatic analyses. Phylogeny of Onagraceae was inferred using maximum-likelihood (ML) and Bayesian inference (BI) methods to understand generic and specific relationships. The results of the present study showed potential values of the complete chloroplast genome sequences in inferring phylogeny of the family and may provide powerful genetic resources for future studies.

## Data Availability

The data presented in the study are deposited in the GenBank repository, accession number MZ326160, and from MZ353628 to MZ353642.

## Footnotes

1 The genus name *Chamaenerion* is accepted in this study according to Sennikov (2011).
