# Corrigendum: Comparative Analysis of Complete Chloroplast Genomes of 13 Species in *Epilobium*, *Circaea*, and *Chamaenerion* and Insights into Phylogenetic Relationships of Onagraceae

**DOI:** 10.3389/fgene.2022.817493

**Published:** 2022-02-15

**Authors:** Yike Luo, Jian He, Rudan Lyu, Jiamin Xiao, Wenhe Li, Min Yao, Linying Pei, Jin Cheng, Jinyu Li, Lei Xie

**Affiliations:** ^1^ School of Ecology and Nature Conservation, Beijing Forestry University, Beijing, China; ^2^ Beijing Engineering Research Center for Landscape Plant, Beijing Forestry University Forest Science Co. Ltd., Beijing, China; ^3^ College of Biological Sciences and Technology, Beijing Forestry University, Beijing, China; ^4^ Beijing Institute of Landscape Architecture, Beijing, China

**Keywords:** chloroplast genome, inversion, Onagraceae, phylogeny, RNA editing, biparental inheritance, IR expansion

In the original article, there was a mistake in **Figure 3** as published. “The number in the IRb region of *Circaea cordata* needed to be corrected.” The corrected **Figure 3** appears below.

**FIGURE 1:**
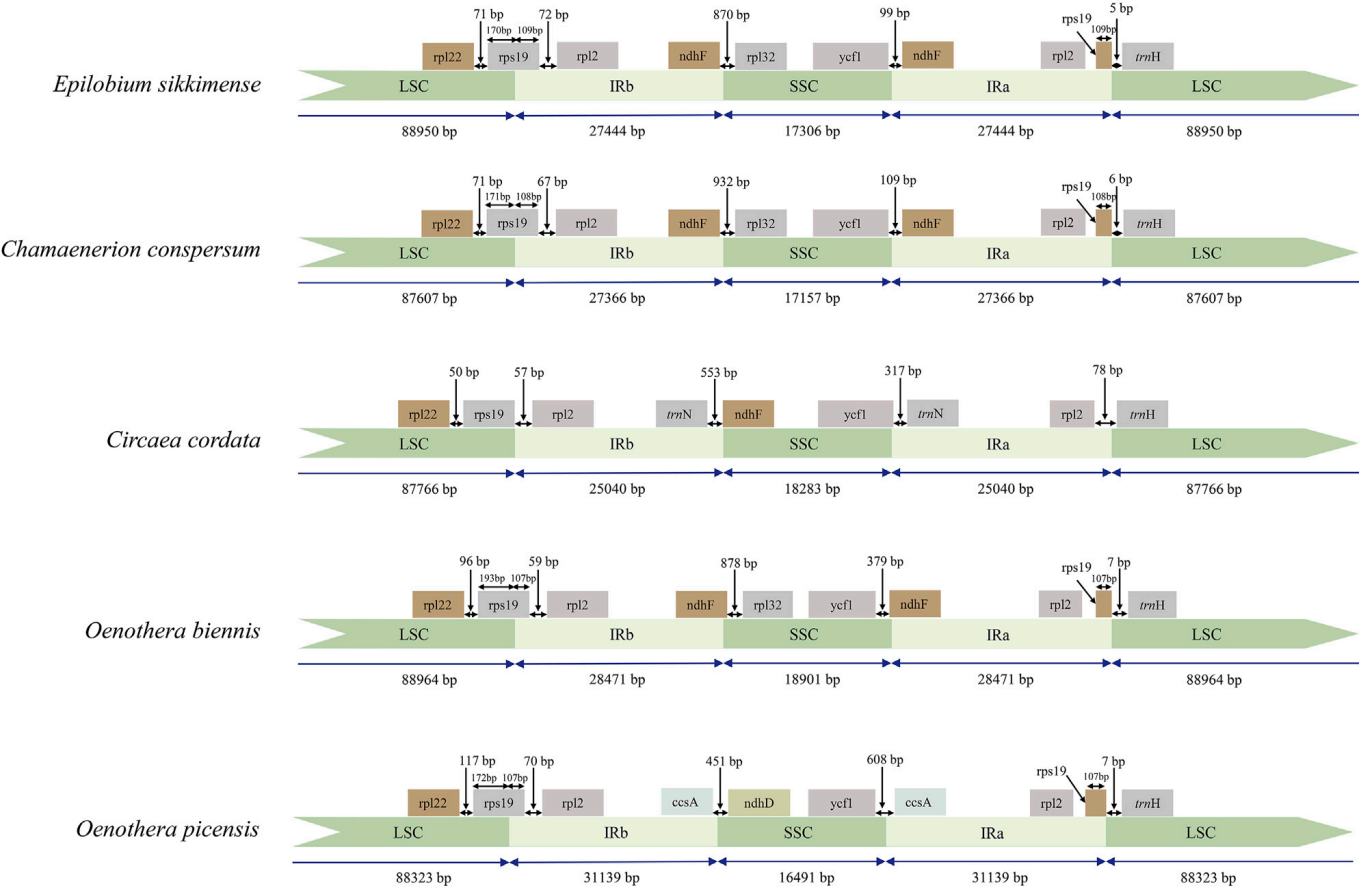
Comparison of the LSC, IR, and SSC boundary regions of representative samples the three newly sequenced genera of Onagraceae and Oenothera samples. IR: inverted repeats; LSC: large single copy; SSC: small single copy.

The authors apologize for this error and state that this does not change the scientific conclusions of the article in any way. The original article has been updated.

