# Supplementary figures and images for: Comparative Analysis of Complete Chloroplast Genomes of 13 Species in Epilobium, Circaea, and Chamaenerion and Insights Into Phylogenetic Relationships of Onagraceae
Source: Front Genet. 2021 Nov 4;12:730495. doi: 10.3389/fgene.2021.730495 (PMC8600051; doi:10.3389/fgene.2021.730495)

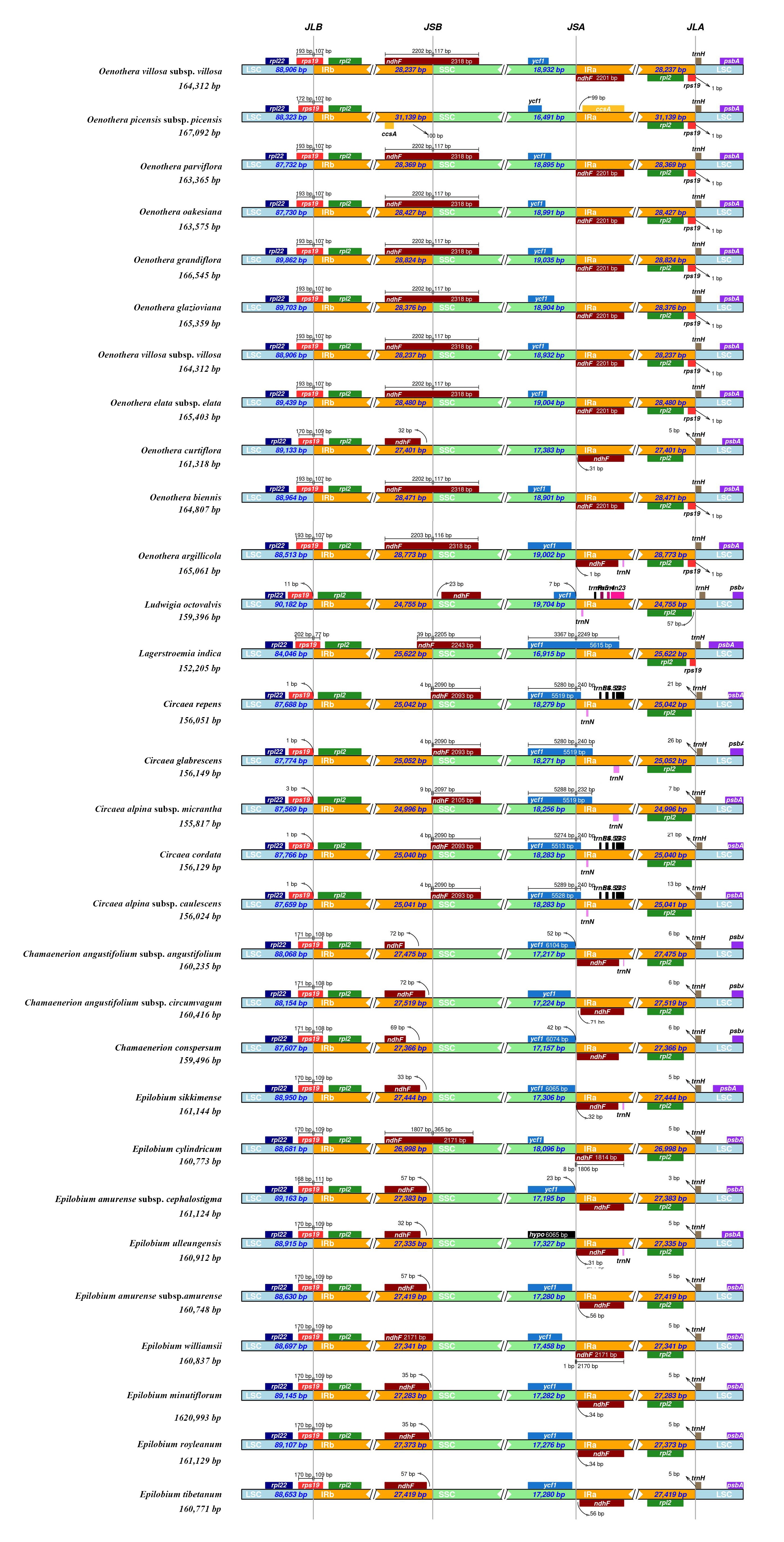

Supplement: Supplementary file 1 [file Image3.JPEG]

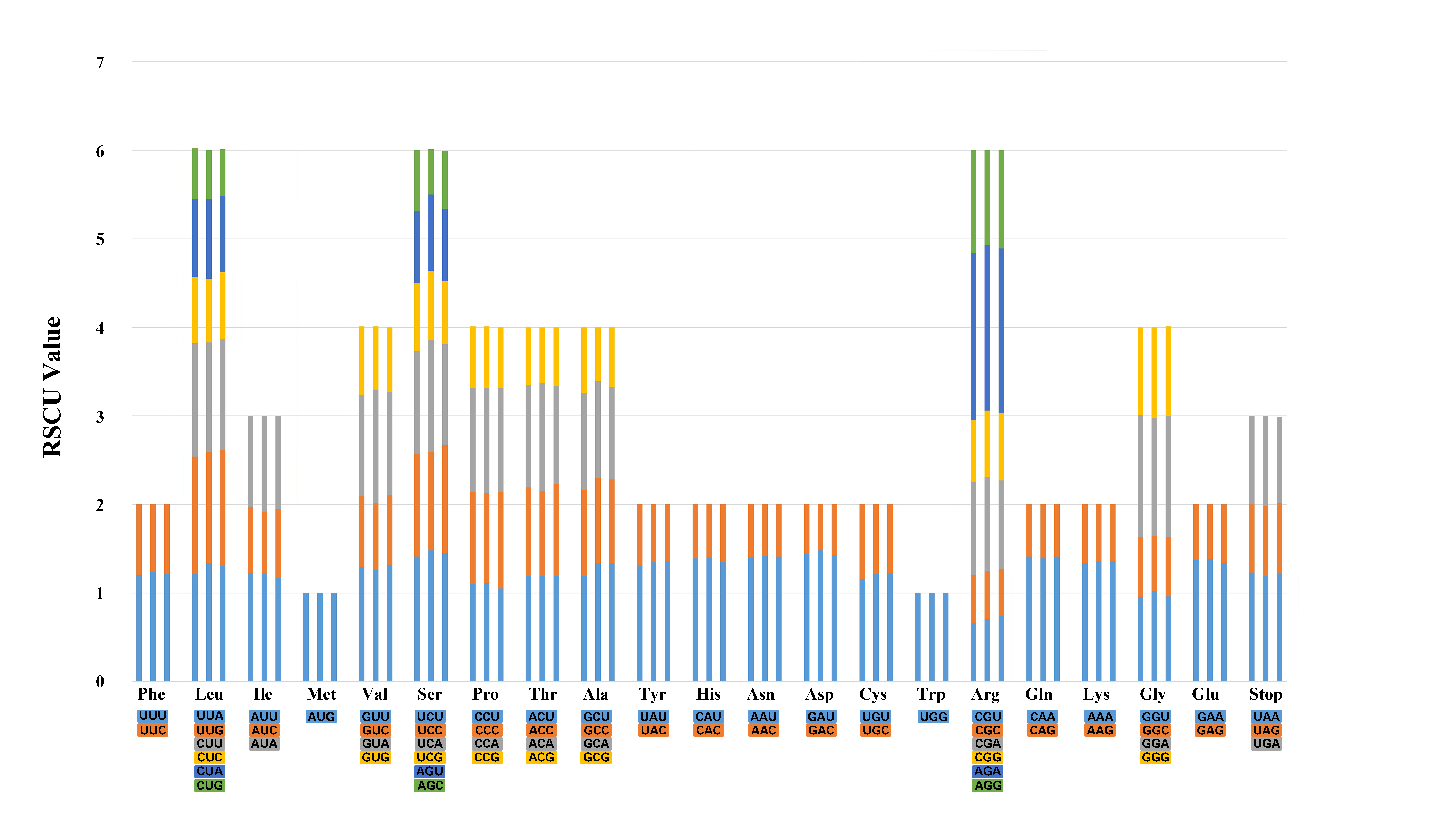

Supplement: Supplementary file 3 [file Image1.JPEG]

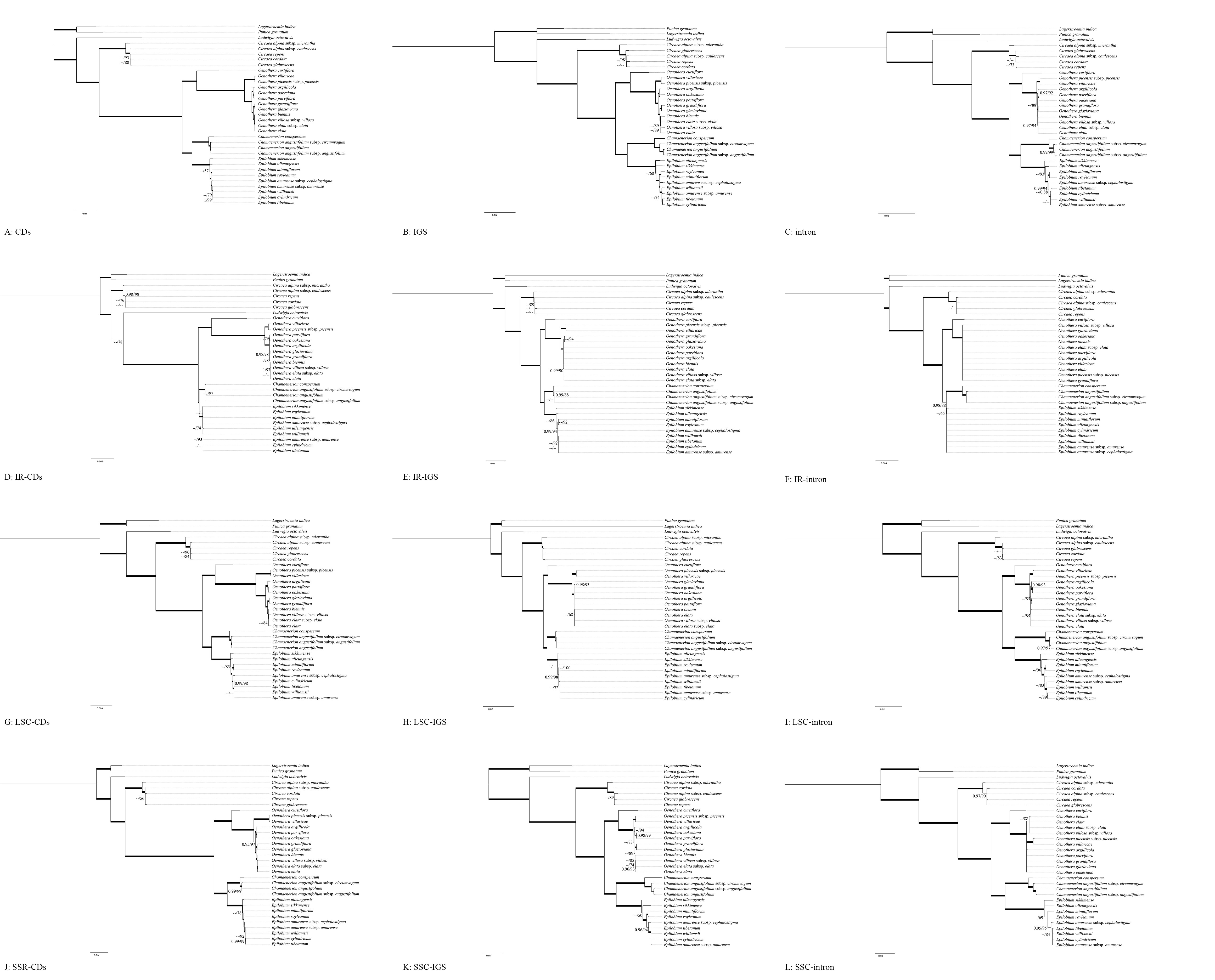

Supplement: Supplementary file 4 [file Image4.JPEG]

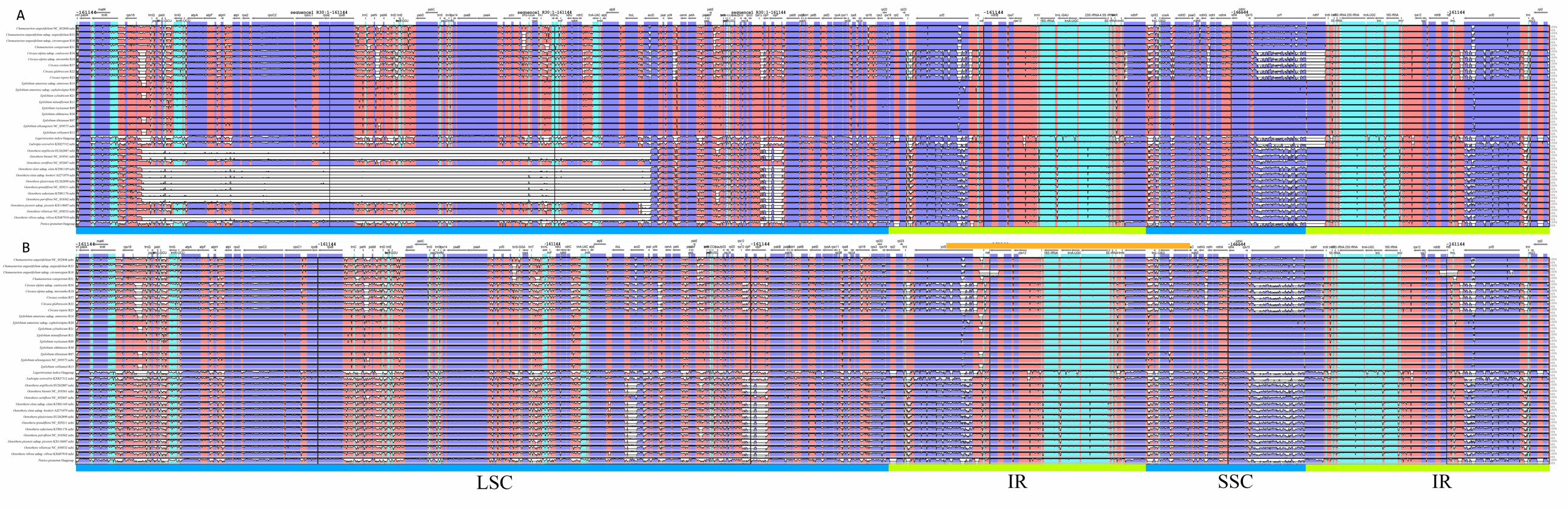

Supplement: Supplementary file 5 [file Image2.JPEG]
