# Supplementary material for: Comparative Analysis of Complete Chloroplast Genomes of 13 Species in Epilobium, Circaea, and Chamaenerion and Insights Into Phylogenetic Relationships of Onagraceae
Source: Front Genet. 2021 Nov 4;12:730495. doi: 10.3389/fgene.2021.730495 (PMC8600051; doi:10.3389/fgene.2021.730495)
Supplement: Supplementary file 2 [file Table1.DOCX]

| **TABLE S1** Sample information pertaining to the present study. | | | | | |
| --- | --- | --- | --- | --- | --- |
| Family | Species | Collecting site | Voucher number | Colleting date | GeneBank accession |
| Onagraceae | *Chamaenerion angustifolium* subsp. *angustifolium* | Litang, Sichuan, China | L. Xie 20200806007 (BJFC) | 6 August 2020 | MZ353639 |
| Onagraceae | *C. angustifolium* subsp. *circumvagum* | Zayu, Xizang, China | Y.K. Luo et al. FB119951 (BJFC) | 2 August 2020 | MZ353632 |
| Onagraceae | *C. conspersum* | Zayu, Xizang, China | Y.K. Luo et al. FB119919 (BJFC) | 2 August 2020 | MZ353638 |
| Onagraceae | *Circaea alpina* subsp. *caulescens* | Changping, Beijing, China | L. Xie 2019082104 (BJFC) | 21 August 2020 | MZ353641 |
| Onagraceae | *C. alpina* subsp. *micrantha* | Zayu, Xizang, China | Y.K. Luo et al. FB101007 (BJFC) | 3 August 2020 | MZ353628 |
| Onagraceae | *C. cordata* | Zayu, Xizang, China | Y.K. Luo et al. FB101069 (BJFC) | 5 August 2020 | MZ353640 |
| Onagraceae | *C. glabrescens* | Emei, Sichuan, China | L. Xie et al. 2018081606 (BJFC) | 16 August 2020 | MZ353635 |
| Onagraceae | *C. repens* | Xinduqiao, Sichuan, China | L. Xie et al. 2018081608 (BJFC) | 17 August 2020 | MZ353636 |
| Onagraceae | *Epilobium amurense* subsp. *amurense* | Zayu, Xizang, China | Y.K. Luo et al. FB119916(BJFC) | 1 August 2020 | MZ353631 |
| Onagraceae | *E. amurense* subsp. *cephalostigma* | Yanqing, Beijing, China | L. Xie 20200916003 (BJFC) | 16 September 2020 | MZ353633 |
| Onagraceae | *E. cylindricum* | Luding, Sichuan, China | L. Xie et al. 2018081210 (BJFC) | 12 August 2020 | MZ353634 |
| Onagraceae | *E. minutiflorum* | Mainling, Sichuan, China | Y.K. Luo et al. FB119719 (BJFC) | 29 July 2020 | MZ353629 |
| Onagraceae | *E. royleanum* | Batang, Sichuan, China | L. Xie 20200808004 (BJFC) | 8 August 2020 | MZ353642 |
| Onagraceae | *E. sikkimense* | Nyingchi, Xizang, China | Y.K. Luo et al. FB119815 (BJFC) | 30 July 2020 | MZ353637 |
| Onagraceae | *E. tibetanum* | Sichuan, China | L. Xie 2020081003 (BJFC) | 10 August 2020 | MZ326160 |
| Onagraceae | *E. williamsii* | Mainling, Sichuan, China | Y.K. Luo et al. FB119817 (BJFC) | 30 July 2020 | MZ353630 |
| Lythraceae | *Lagerstroemia indica** |  |  |  | NC_030484 |
| Lythraceae | *Punica granatum** |  |  |  | MK635347 (Yan, 2019) |
| Onagraceae | *Chamaenerion angustifolium** |  |  |  | MN481508（Li, 2019） |
| Onagraceae | *Epilobium ulleungensis** |  |  |  | MH198310 (Yang et al., 2018) |
| Onagraceae | *Ludwigia octovalvis** |  |  |  | KX827312 (Liu et al., 2016) |
| Onagraceae | *Oenothera argillicola** |  |  |  | EU262887 (Greiner, 2008) |
| Onagraceae | *O. biennis** |  |  |  | EU262889 (Greiner, 2008) |
| Onagraceae | *O. curtiflora** |  |  |  | NC_052847 |
| Onagraceae | *O. elata* subsp. *elata** |  |  |  | KT881169 (Massouh, 2016) |
| Onagraceae | *O. elata* subsp. *hookeri** |  |  |  | AJ271079 (Hupfer, 2008) |
| Onagraceae | *O. glazioviana** |  |  |  | EU262890 (Greiner, 2008) |
| Onagraceae | *O. grandiflora** |  |  |  | NC_029211 (Massouh, 2016) |
| Onagraceae | *O. oakesiana** |  |  |  | KT881176 (Massouh, 2016) |
| Onagraceae | *O. parviflora** |  |  |  | EU262891 (Greiner, 2008) |
| Onagraceae | *O. picensis* subsp. *picensis** |  |  |  | KX118607 |
| Onagraceae | *O. villaricae ** |  |  |  | NC_030532 |
| Onagraceae | *O. villosa* subsp. *villosa** |  |  |  | KX687910 (Sobanski, 2019) |

*Downloaded from GenBank.
