# Supplementary material for: Comparative Analysis of Complete Chloroplast Genomes of 13 Species in Epilobium, Circaea, and Chamaenerion and Insights Into Phylogenetic Relationships of Onagraceae
Source: Front Genet. 2021 Nov 4;12:730495. doi: 10.3389/fgene.2021.730495 (PMC8600051; doi:10.3389/fgene.2021.730495)
Supplement: Supplementary file 6 [file Table3.DOCX]

**TABLE S3** Prediction of RNA editing by the PREP-cp program.

1. ***Epilobium***

| Gene | Nucleotide position | Triplet position | Bases | Codon change | Amino acid change |
| --- | --- | --- | --- | --- | --- |
| accD | 26 | 2 | C-T | ACG-ATG | T-M |
|  | 761 | 2 | C-T | ACT-ATT | T-I |
|  | 1538 | 2 | C-T | CCT-CTT | P-L |
| atpA | 791 | 2 | C-T | CCC-CTC | P-L |
| atpF | 92 | 2 | C-T | CCA-CTA | P-L |
| atpI | 629 | 2 | C-T | TCA-TTA | S-L |
| ccsA | 334 | 1 | C-T | CTT-TTT | L-F |
| clpP | 353 | 2 | C-T | TCC-TTC | S-F |
| matK | 1204 | 1 | C-T | CGG-TGG | R-W |
|  | 1214 | 2 | C-T | TCA-TTA | S-L |
|  | 1273 | 1 | C-T | CAC-TAC | H-Y |
| ndhB | 28 | 1 | C-T | CTC-TTC | L-F |
|  | 74 | 2 | C-T | CCT-CTT | P-L |
|  | 149 | 2 | C-T | TCA-TTA | S-L |
|  | 467 | 2 | C-T | CCA-CTA | P-L |
|  | 586 | 1 | C-T | CAT-TAT | H-Y |
|  | 611 | 2 | C-T | TCA-TTA | S-L |
|  | 737 | 2 | C-T | CCA-CTA | P-L |
|  | 746 | 2 | C-T | TCT-TTT | S-F |
|  | 830 | 2 | C-T | TCA-TTA | S-L |
|  | 836 | 2 | C-T | TCA-TTA | S-L |
|  | 1255 | 1 | C-T | CAT-TAT | H-Y |
|  | 1481 | 2 | C-T | CCA-CTA | P-L |
| ndhD | 29 | 2 | C-T | ACG-ATG | T-M |
|  | 212 | 2 | C-T | ACC-ATC | T-I |
|  | 905 | 2 | C-T | TCA-TTA | S-L |
|  | 914 | 2 | C-T | CCT-CTT | P-L |
|  | 1325 | 2 | C-T | TCA-TTA | S-L |
|  | 1432 | 1 | C-T | CTT-TTT | L-F |
| ndhF | 13 | 1 | C-T | CTT-TTT | L-F |
|  | 112 | 1 | C-T | CAC-TAC | H-Y |
|  | 439 | 1 | C-T | CTT-TTT | L-F |
|  | 2075 | 2 | C-T | TCT-TTT | S-F |
| ndhG | 314 | 2 | C-T | ACA-ATA | T-I |
| psaI | 67 | 1 | C-T | CCC-TCC | P-S |
|  | 149 | 2 | C-T | TCT-TTT | S-F |
| psbF | 77 | 2 | C-T | TCT-TTT | S-F |
| rpoB | 338 | 2 | C-T | TCT-TTT | S-F |
|  | 551 | 2 | C-T | TCA-TTA | S-L |
|  | 566 | 2 | C-T | TCG-TTG | S-L |
|  | 917 | 2 | C-T | ACG-ATG | T-M |
| rpoC1 | 41 | 2 | C-T | TCA-TTA | S-L |
|  | 338 | 2 | C-T | GCA-GTA | A-V |
|  | 1171 | 1 | C-T | CCA-TCA | P-S |
| rpoC2 | 1135 | 1 | C-T | CTT-TTT | L-F |
|  | 1726 | 1 | C-T | CTT-TTT | L-F |
|  | 3100 | 1 | C-T | CGC-TGC | R-C |
| rps14 | 80 | 2 | C-T | TCA-TTA | S-L |
|  | 149 | 2 | C-T | TCA-TTA | S-L |
| rps16 | 47 | 2 | C-T | CCG-CTG | P-L |

1. ***Circaea***

| Gene | Nucleotide position | Triplet position | Bases | Codon change | Amino acid change |
| --- | --- | --- | --- | --- | --- |
| accD | 1445 | 2 | C-T | CCT-CTT | P-L |
| atpA | 791 | 2 | C-T | CCC-CTC | P-L |
| atpF | 92 | 2 | C-T | CCA-CTA | P-L |
| atpI | 23 | 2 | C-T | ACC-ATC | T-I |
|  | 629 | 2 | C-T | TCA-TTA | S-L |
| ccsA | 89 | 2 | C-T | TCA-TTA | S-L |
| matK | 650 | 2 | C-T | GCA-GTA | A-V |
|  | 1198 | 1 | C-T | CGG-TGG | R-W |
|  | 1208 | 2 | C-T | TCA-TTA | S-L |
| ndhB | 28 | 1 | C-T | CTC-TTC | L-F |
|  | 74 | 2 | C-T | CCT-CTT | P-L |
|  | 149 | 2 | C-T | TCA-TTA | S-L |
|  | 467 | 2 | C-T | CCA-CTA | P-L |
|  | 586 | 1 | C-T | CAT-TAT | H-Y |
|  | 611 | 2 | C-T | TCA-TTA | S-L |
|  | 737 | 2 | C-T | CCA-CTA | P-L |
|  | 746 | 2 | C-T | TCT-TTT | S-F |
|  | 830 | 2 | C-T | TCA-TTA | S-L |
|  | 836 | 2 | C-T | TCA-TTA | S-L |
|  | 1255 | 1 | C-T | CAT-TAT | H-Y |
|  | 1481 | 2 | C-T | CCA-CTA | P-L |
| ndhD | 29 | 2 | C-T | ACG-ATG | T-M |
|  | 212 | 2 | C-T | ACC-ATC | T-I |
|  | 905 | 2 | C-T | TCA-TTA | S-L |
|  | 914 | 2 | C-T | CCT-CTT | P-L |
|  | 1325 | 2 | C-T | TCA-TTA | S-L |
|  | 1432 | 1 | C-T | CTT-TTT | L-F |
| ndhF | 13 | 1 | C-T | CTT-TTT | L-F |
|  | 439 | 1 | C-T | CTT-TTT | L-F |
|  | 2072 | 2 | C-T | TCT-TTT | S-F |
| ndhG | 314 | 2 | C-T | ACA-ATA | T-I |
| psaI | 67 | 2 | C-T | CCC-TCC | P-S |
|  | 149 | 2 | C-T | TCT-TTT | S-F |
| psbB | 305 | 2 | C-T | GCT-GTT | A-V |
| psbF | 77 | 2 | C-T | TCT-TTT | S-F |
| rpoA | 1022 | 2 | C-T | TCA-TTA | S-L |
| rpoB | 338 | 2 | C-T | TCT-TTT | S-F |
|  | 551 | 2 | C-T | TCA-TTA | S-L |
|  | 566 | 2 | C-T | TCG-TTG | S-L |
|  | 917 | 2 | C-T | ACG-ATG | T-M |
|  | 973 | 1 | C-T | CTC-TTC | L-F |
| rps14 | 80 | 2 | C-T | TCA-TTA | S-L |
|  | 149 | 2 | C-T | TCA-TTA | S-L |

1. ***Chamaenerion***

| Gene | Nucleotide position | Triplet position | Bases | Codon change | Amino acid change |
| --- | --- | --- | --- | --- | --- |
| accD | 26 | 2 | C-T | ACG-ATG | T-M |
| atpA | 791 | 2 | C-T | CCC-CTC | P-L |
| atpF | 92 | 2 | C-T | CCA-CTA | P-L |
| atpI | 629 | 2 | C-T | TCA-TTA | S-L |
| ccsA | 89 | 2 | C-T | TCA-TTA | S-L |
|  | 334 | 1 | C-T | CTT-TTT | L-F |
| clpP | 353 | 2 | C-T | TCC-TTC | S-F |
| matK | 1189 | 1 | C-T | CGG-TGG | R-W |
|  | 1199 | 2 | C-T | TCA-TTA | S-L |
|  | 1258 | 1 | C-T | CAC-TAC | H-Y |
| ndhB | 28 | 1 | C-T | CTC-TTC | L-F |
|  | 74 | 2 | C-T | CCT-CTT | P-L |
|  | 149 | 2 | C-T | TCA-TTA | S-L |
|  | 467 | 2 | C-T | CCA-CTA | P-L |
|  | 586 | 1 | C-T | CAT-TAT | H-Y |
|  | 611 | 2 | C-T | TCA-TTA | S-L |
|  | 737 | 2 | C-T | CCA-CTA | P-L |
|  | 746 | 2 | C-T | TCT-TTT | S-F |
|  | 830 | 2 | C-T | TCA-TTA | S-L |
|  | 836 | 2 | C-T | TCA-TTA | S-L |
|  | 1255 | 1 | C-T | CAT-TAT | H-Y |
|  | 1481 | 2 | C-T | CCA-CTA | P-L |
| ndhD | 92 | 2 | C-T | ACC-ATC | T-I |
|  | 290 | 2 | C-T | TCA-TTA | S-L |
|  | 785 | 2 | C-T | TCA-TTA | S-L |
|  | 794 | 2 | C-T | CCT-CTT | P-L |
|  | 1205 | 2 | C-T | TCA-TTA | S-L |
|  | 1312 | 1 | C-T | CTT-TTT | L-F |
| ndhF | 13 | 1 | C-T | CTT-TTT | L-F |
|  | 112 | 1 | C-T | CAC-TAC | H-Y |
|  | 439 | 1 | C-T | CTT-TTT | L-F |
|  | 2075 | 2 | C-T | TCT-TTT | S-F |
| ndhG | 314 | 2 | C-T | ACA-ATA | T-I |
| psaI | 149 | 2 | C-T | TCT-TTT | S-F |
| psbF | 77 | 2 | C-T | TCT-TTT | S-F |
| rpoB | 338 | 2 | C-T | TCT-TTT | S-F |
|  | 551 | 2 | C-T | TCA-TTA | S-L |
|  | 566 | 2 | C-T | TCG-TTG | S-L |
|  | 917 | 2 | C-T | ACG-ATG | T-M |
| rpoC1 | 41 | 2 | C-T | TCA-TTA | S-L |
|  | 338 | 2 | C-T | GCA-GTA | A-V |
|  | 1171 | 1 | C-T | CCA-TCA | P-S |
| rpoC2 | 1135 | 1 | C-T | CTT-TTT | L-F |
|  | 1726 | 1 | C-T | CTT-TTT | L-F |
|  | 3041 | 2 | C-T | TCT-TTT | S-F |
|  | 3100 | 1 | C-T | CGC-TGC | R-C |
| rps14 | 80 | 2 | C-T | TCA-TTA | S-L |
|  | 149 | 2 | C-T | TCA-TTA | S-L |

1. **Summary**

| Genus  Amino  acid change | *Epilobium* | | *Circaea* | | *Chamaenerion* | |
| --- | --- | --- | --- | --- | --- | --- |
|  | Numbers | Percentage | Numbers | Percentage | Numbers | Percentage |
| A-V | 1 | 2% | 2 | 5% | 1 | 2% |
| H-Y | 4 | 8% | 2 | 5% | 4 | 8% |
| L-F | 7 | 14% | 5 | 12% | 7 | 15% |
| P-L | 9 | 18% | 8 | 19% | 7 | 15% |
| P-S | 2 | 4% | 1 | 2% | 1 | 2% |
| R-C | 1 | 2% | - | - | 1 | 2% |
| R-W | 1 | 2% | 1 | 2% | 1 | 2% |
| S-F | 6 | 12% | 5 | 12% | 7 | 15% |
| S-L | 13 | 26% | 14 | 33% | 15 | 31% |
| T-I | 3 | 6% | 3 | 7% | 2 | 4% |
| T-M | 3 | 6% | 2 | 5% | 2 | 4% |
