# Supplementary material for: Comparative Analysis of Complete Chloroplast Genomes of 13 Species in Epilobium, Circaea, and Chamaenerion and Insights Into Phylogenetic Relationships of Onagraceae
Source: Front Genet. 2021 Nov 4;12:730495. doi: 10.3389/fgene.2021.730495 (PMC8600051; doi:10.3389/fgene.2021.730495)
Supplement: Supplementary file 7 [file Table4.DOCX]

**TABLE S4a -** Base composition in the *Epilobium tibetanum* plastid genome.

|  | **T %** | **C %** | **A%** | **G%** | **Length (bp)** |
| --- | --- | --- | --- | --- | --- |
| **Total** | 31.4 | 19.4 | 30.5 | 18.7 | 160771 |
| **LSC** | 32.5 | 18.5 | 31.2 | 17.8 | 88653 |
| **SSC** | 34.0 | 17.6 | 32.9 | 15.5 | 17280 |
| **IR** | 27.6 | 20.4 | 29.7 | 22.3 | 27419 |
| **tRNA** | 22.3 | 27.7 | 22.3 | 27.7 | 9050 |
| **rRNA** | 22.6 | 26.9 | 24.0 | 26.5 | 2783 |
| **Protein Coding genes** | 30.2 | 20.8 | 29.0 | 19.9 | 92050 |
| **1st Position** | 31.5 | 18.8 | 31.0 | 18.7 | 53591 |
| **2nd position** | 31.4 | 19.6 | 30.0 | 19.0 | 53590 |
| **3rd position** | 31.1 | 19.8 | 30.5 | 18.6 | 53590 |

**TABLE S4b -** Base composition in the *E. williamsii* plastid genome.

|  | **T %** | **C %** | **A%** | **G%** | **Length (bp)** |
| --- | --- | --- | --- | --- | --- |
| **Total** | 31.4 | 19.4 | 30.5 | 18.7 | 160837 |
| **LSC** | 32.5 | 18.5 | 31.2 | 17.8 | 88697 |
| **SSC** | 34.0 | 17.7 | 32.8 | 15.5 | 17306 |
| **IR** | 27.7 | 20.4 | 29.6 | 22.3 | 27341 |
| **tRNA** | 22.7 | 26.9 | 23.8 | 26.6 | 2783 |
| **rRNA** | 22.3 | 27.7 | 22.3 | 27.7 | 9050 |
| **Protein Coding genes** | 30.2 | 20.8 | 29.0 | 19.9 | 92134 |
| **1st Position** | 31.5 | 18.9 | 30.6 | 19.0 | 53613 |
| **2nd position** | 30.9 | 20.4 | 29.6 | 19.1 | 53612 |
| **3rd position** | 31.7 | 18.9 | 31.3 | 18.1 | 53612 |

**TABLE S4c -** Base composition in the *E. sikkimense* plastid genome.

|  | **T %** | **C %** | **A%** | **G%** | **Length (bp)** |
| --- | --- | --- | --- | --- | --- |
| **Total** | 31.4 | 19.4 | 30.5 | 18.7 | 161144 |
| **LSC** | 32.5 | 18.5 | 31.2 | 17.8 | 88950 |
| **SSC** | 34.0 | 17.7 | 32.8 | 15.5 | 17306 |
| **IR** | 27.6 | 20.4 | 29.6 | 22.3 | 27373 |
| **tRNA** | 22.7 | 26.9 | 23.8 | 26.6 | 2783 |
| **rRNA** | 22.3 | 27.7 | 22.3 | 27.7 | 9050 |
| **Protein Coding genes** | 30.2 | 20.8 | 29.0 | 19.9 | 92212 |
| **1st Position** | 31.5 | 18.9 | 30.8 | 18.8 | 53715 |
| **2nd position** | 31.3 | 19.5 | 30.5 | 18.7 | 53715 |
| **3rd position** | 31.3 | 19.8 | 30.2 | 18.7 | 53714 |

**TABLE S4d -** Base composition in the *E. royleanum* plastid genome.

|  | **T %** | **C %** | **A%** | **G%** | **Length (bp)** |
| --- | --- | --- | --- | --- | --- |
| **Total** | 31.3 | 19.4 | 30.5 | 18.8 | 161129 |
| **LSC** | 32.5 | 18.5 | 31.2 | 17.8 | 89107 |
| **SSC** | 33.9 | 17.7 | 32.8 | 15.5 | 17276 |
| **IR** | 27.6 | 20.4 | 29.6 | 22.3 | 27373 |
| **tRNA** | 22.7 | 26.9 | 23.8 | 26.6 | 2783 |
| **rRNA** | 22.3 | 27.7 | 22.3 | 27.7 | 9050 |
| **Protein Coding genes** | 30.2 | 20.8 | 29.0 | 19.9 | 93205 |
| **1st Position** | 30.9 | 19.6 | 30.7 | 18.8 | 53710 |
| **2nd position** | 31.7 | 18.8 | 31.2 | 18.4 | 53710 |
| **3rd position** | 31.4 | 19.9 | 29.6 | 19.1 | 53709 |

**TABLE S4e -** Base composition in the *E. minutiflorum* plastid genome.

|  | **T %** | **C %** | **A%** | **G%** | **Length (bp)** |
| --- | --- | --- | --- | --- | --- |
| **Total** | 31.3 | 19.4 | 30.5 | 18.7 | 160993 |
| **LSC** | 32.5 | 18.5 | 31.3 | 17.8 | 89145 |
| **SSC** | 33.9 | 17.7 | 32.8 | 15.5 | 17282 |
| **IR** | 27.7 | 20.4 | 29.6 | 22.3 | 27283 |
| **tRNA** | 22.7 | 26.9 | 23.8 | 26.6 | 2783 |
| **rRNA** | 22.3 | 27.7 | 22.3 | 27.7 | 9050 |
| **Protein Coding genes** | 30.2 | 20.8 | 29.0 | 19.9 | 91954 |
| **1st Position** | 31.8 | 19.2 | 30.7 | 18.2 | 53665 |
| **2nd position** | 31.1 | 19.4 | 30.3 | 19.1 | 53664 |
| **3rd position** | 31.1 | 19.5 | 30.5 | 18.9 | 53664 |

**TABLE S4f -** Base composition in the *E. cylindricum* plastid genome.

|  | **T %** | **C %** | **A%** | **G%** | **Length (bp)** |
| --- | --- | --- | --- | --- | --- |
| **Total** | 31.4 | 19.4 | 30.5 | 18.7 | 160773 |
| **LSC** | 32.5 | 18.5 | 31.2 | 17.7 | 88681 |
| **SSC** | 33.9 | 17.7 | 32.9 | 15.5 | 17254 |
| **IR** | 27.6 | 20.4 | 29.7 | 22.3 | 27419 |
| **tRNA** | 22.7 | 26.9 | 23.8 | 26.6 | 2783 |
| **rRNA** | 22.3 | 27.7 | 22.3 | 27.7 | 9050 |
| **Protein Coding genes** | 30.2 | 20.9 | 29.0 | 19.9 | 91816 |
| **1st Position** | 31.1 | 19.8 | 30.0 | 19.1 | 53591 |
| **2nd position** | 31.7 | 19.3 | 30.8 | 18.2 | 53591 |
| **3rd position** | 31.3 | 19.1 | 30.7 | 18.9 | 53591 |

**TABLE S4g -** Base composition in the *E. amurense* subsp. *cephalostigma* plastid genome.

|  | **T %** | **C %** | **A%** | **G%** | **Length (bp)** |
| --- | --- | --- | --- | --- | --- |
| **Total** | 31.3 | 19.4 | 30.5 | 18.7 | 161124 |
| **LSC** | 32.5 | 18.6 | 31.2 | 17.8 | 88630 |
| **SSC** | 33.8 | 17.8 | 32.9 | 15.5 | 17195 |
| **IR** | 27.6 | 20.4 | 29.7 | 22.3 | 27383 |
| **tRNA** | 22.7 | 26.9 | 23.8 | 26.6 | 2783 |
| **rRNA** | 22.3 | 27.7 | 22.3 | 27.7 | 9050 |
| **Protein Coding genes** | 30.2 | 20.8 | 29.0 | 19.9 | 92182 |
| **1st Position** | 31.1 | 18.9 | 31.1 | 18.9 | 53708 |
| **2nd position** | 31.1 | 19.4 | 30.7 | 18.8 | 53708 |
| **3rd position** | 31.8 | 19.9 | 29.8 | 18.5 | 53708 |

**TABLE S4h -** Base composition in the *E. amurense* subsp. *amurense* plastid genome.

|  | **T %** | **C %** | **A%** | **G%** | **Length (bp)** |
| --- | --- | --- | --- | --- | --- |
| **Total** | 31.3 | 19.4 | 30.5 | 18.7 | 160748 |
| **LSC** | 32.5 | 18.6 | 31.2 | 17.8 | 88630 |
| **SSC** | 34.0 | 17.7 | 32.9 | 15.5 | 17280 |
| **IR** | 27.6 | 20.4 | 29.7 | 22.3 | 27419 |
| **tRNA** | 22.7 | 26.9 | 23.8 | 26.6 | 2783 |
| **rRNA** | 22.3 | 27.7 | 22.3 | 27.7 | 9050 |
| **Protein Coding genes** | 30.2 | 20.8 | 29.0 | 19.9 | 92206 |
| **1st Position** | 31.6 | 19.3 | 30.8 | 18.3 | 53583 |
| **2nd position** | 31.7 | 18.8 | 30.6 | 18.9 | 53583 |
| **3rd position** | 30.7 | 20.1 | 30.0 | 19.1 | 53582 |

**TABLE S4i -** Base composition in the *Circaea alpina* subsp. *caulescens* plastid genome.

|  | **T %** | **C %** | **A%** | **G%** | **Length (bp)** |
| --- | --- | --- | --- | --- | --- |
| **Total** | 31.6 | 19.2 | 30.7 | 18.6 | 156024 |
| **LSC** | 32.9 | 18.3 | 31.3 | 17.4 | 87659 |
| **SSC** | 33.7 | 16.7 | 34.1 | 15.5 | 18283 |
| **IR** | 28.4 | 20.8 | 28.3 | 22.5 | 25041 |
| **tRNA** | 22.9 | 26.6 | 23.6 | 26.9 | 2554 |
| **rRNA** | 22.4 | 27.6 | 22.4 | 27.6 | 9050 |
| **Protein Coding genes** | 30.2 | 20.6 | 29.3 | 19.8 | 90590 |
| **1st Position** | 31.9 | 19.1 | 30.4 | 18.6 | 52008 |
| **2nd position** | 31.0 | 19.3 | 31.0 | 18.7 | 52008 |
| **3rd position** | 31.6 | 19.3 | 30.7 | 18.4 | 52008 |

**TABLE S4j -** Base composition in the *C. repens* plastid genome.

|  | **T %** | **C %** | **A%** | **G%** | **Length (bp)** |
| --- | --- | --- | --- | --- | --- |
| **Total** | 31.6 | 19.2 | 30.7 | 18.6 | 156051 |
| **LSC** | 32.9 | 18.3 | 31.3 | 17.4 | 87688 |
| **SSC** | 33.7 | 16.7 | 34.1 | 15.5 | 18279 |
| **IR** | 28.4 | 20.8 | 28.3 | 22.4 | 25042 |
| **tRNA** | 22.7 | 26.9 | 23.8 | 26.6 | 2783 |
| **rRNA** | 22.3 | 27.7 | 22.3 | 27.7 | 9050 |
| **Protein Coding genes** | 30.3 | 20.6 | 29.3 | 19.8 | 90593 |
| **1st Position** | 31.2 | 19.3 | 30.5 | 19.0 | 52017 |
| **2nd position** | 31.8 | 19.7 | 30.5 | 18.1 | 52017 |
| **3rd position** | 31.7 | 18.5 | 31.1 | 18.6 | 52017 |

**TABLE S4k -** Base composition in the *C. glabrescens* plastid genome.

|  | **T %** | **C %** | **A%** | **G%** | **Length (bp)** |
| --- | --- | --- | --- | --- | --- |
| **Total** | 31.6 | 19.2 | 30.7 | 18.5 | 156149 |
| **LSC** | 33.0 | 18.3 | 31.3 | 17.4 | 87774 |
| **SSC** | 33.7 | 16.7 | 34.1 | 15.5 | 18271 |
| **IR** | 28.4 | 20.8 | 28.3 | 22.4 | 25052 |
| **tRNA** | 22.7 | 26.9 | 23.8 | 26.6 | 2783 |
| **rRNA** | 22.3 | 27.7 | 22.3 | 27.7 | 9050 |
| **Protein Coding genes** | 30.3 | 20.6 | 29.3 | 19.8 | 90593 |
| **1st Position** | 31.5 | 18.9 | 30.5 | 19.0 | 52050 |
| **2nd position** | 31.1 | 19.7 | 30.8 | 18.4 | 52050 |
| **3rd position** | 32.1 | 18.9 | 30.8 | 18.2 | 52049 |

**TABLE S4l -** Base composition in the *C. alpina* subsp. *micrantha* plastid genome.

|  | **T %** | **C %** | **A%** | **G%** | **Length (bp)** |
| --- | --- | --- | --- | --- | --- |
| **Total** | 31.5 | 19.2 | 30.6 | 18.6 | 155817 |
| **LSC** | 32.9 | 18.4 | 31.3 | 17.5 | 87569 |
| **SSC** | 33.7 | 16.8 | 34.0 | 15.5 | 18256 |
| **IR** | 28.4 | 20.9 | 28.3 | 22.5 | 24996 |
| **tRNA** | 22.7 | 26.9 | 23.8 | 26.6 | 2783 |
| **rRNA** | 22.3 | 27.7 | 22.3 | 27.7 | 9050 |
| **Protein Coding genes** | 30.2 | 20.6 | 29.3 | 19.8 | 90446 |
| **1st Position** | 31.3 | 19.5 | 30.9 | 18.4 | 51939 |
| **2nd position** | 31.7 | 19.2 | 30.0 | 19.1 | 51939 |
| **3rd position** | 31.6 | 19.0 | 31.1 | 18.3 | 51939 |

**TABLE S4m -** Base composition in the *C. cordata* plastid genome.

|  | **T %** | **C %** | **A%** | **G%** | **Length (bp)** |
| --- | --- | --- | --- | --- | --- |
| **Total** | 31.5 | 19.2 | 30.7 | 18.6 | 156129 |
| **LSC** | 32.9 | 18.3 | 31.3 | 17.4 | 87766 |
| **SSC** | 33.6 | 16.7 | 34.1 | 15.6 | 18283 |
| **IR** | 28.4 | 20.8 | 28.3 | 22.5 | 25040 |
| **tRNA** | 22.7 | 26.9 | 23.8 | 26.6 | 2783 |
| **rRNA** | 22.3 | 27.7 | 22.3 | 27.7 | 9050 |
| **Protein Coding genes** | 30.3 | 20.6 | 29.3 | 19.8 | 90518 |
| **1st Position** | 31.3 | 19.5 | 30.9 | 18.4 | 52043 |
| **2nd position** | 31.7 | 19.2 | 30.0 | 19.1 | 52043 |
| **3rd position** | 31.6 | 19.0 | 31.1 | 18.3 | 52043 |

**TABLE S4n -** Base composition in the *Chamaenerion angustifolium* plastid genome.

|  | **T %** | **C %** | **A%** | **G%** | **Length (bp)** |
| --- | --- | --- | --- | --- | --- |
| **Total** | 31.4 | 19.4 | 30.4 | 18.8 | 160235 |
| **LSC** | 32.6 | 18.6 | 31 | 17.8 | 88068 |
| **SSC** | 34.3 | 17.5 | 32.6 | 15.6 | 17217 |
| **IR** | 27.8 | 20.5 | 29.6 | 22.2 | 27475 |
| **tRNA** | 22.7 | 26.9 | 23.8 | 26.6 | 2783 |
| **rRNA** | 22.3 | 27.7 | 22.3 | 27.7 | 9050 |
| **Protein Coding genes** | 30.3 | 20.8 | 29.0 | 19.9 | 92017 |
| **1st Position** | 31.1 | 19.5 | 30 | 19.4 | 53412 |
| **2nd position** | 31.3 | 19.9 | 30.1 | 18.7 | 53412 |
| **3rd position** | 31.8 | 18.9 | 31.1 | 18.2 | 53411 |

**TABLE S4o -** Base composition in the *C. angustifolium* subsp. *circumvagum* plastid genome.

|  | **T %** | **C %** | **A%** | **G%** | **Length (bp)** |
| --- | --- | --- | --- | --- | --- |
| **Total** | 31.4 | 19.4 | 30.4 | 18.8 | 160416 |
| **LSC** | 32.6 | 18.6 | 31 | 17.8 | 88154 |
| **SSC** | 34.3 | 17.5 | 32.5 | 15.6 | 17224 |
| **IR** | 27.8 | 20.5 | 29.5 | 22.2 | 27519 |
| **tRNA** | 22.7 | 26.9 | 23.8 | 26.6 | 2783 |
| **rRNA** | 22.3 | 27.7 | 22.3 | 27.7 | 9050 |
| **Protein Coding genes** | 30.3 | 20.8 | 29 | 19.9 | 92083 |
| **1st Position** | 32.1 | 19.3 | 30.3 | 18.3 | 53472 |
| **2nd position** | 30.4 | 19.2 | 31 | 19.4 | 53472 |
| **3rd position** | 31.7 | 19.8 | 29.9 | 18.6 | 53472 |

**TABLE S4p -** Base composition in the *C. conspersum* plastid genome.

|  | **T %** | **C %** | **A%** | **G%** | **Length (bp)** |
| --- | --- | --- | --- | --- | --- |
| **Total** | 31.5 | 19.4 | 30.4 | 18.7 | 159496 |
| **LSC** | 32.7 | 18.6 | 31.1 | 17.7 | 87607 |
| **SSC** | 34.2 | 17.6 | 32.6 | 15.5 | 17157 |
| **IR** | 27.8 | 20.5 | 29.5 | 22.2 | 27366 |
| **tRNA** | 22.7 | 26.9 | 23.8 | 26.6 | 2783 |
| **rRNA** | 22.3 | 27.7 | 22.3 | 27.7 | 9050 |
| **Protein Coding genes** | 30.3 | 20.8 | 29 | 19.9 | 91723 |
| **1st Position** | 31.8 | 18.7 | 30.6 | 19 | 53166 |
| **2nd position** | 31.1 | 19.7 | 30.5 | 18.6 | 53165 |
| **3rd position** | 31.5 | 19.8 | 30.1 | 18.6 | 53165 |
